# Oligodendrogenesis increases in hippocampal grey and white matter prior to locomotor or memory impairment in an adult mouse model of tauopathy

**DOI:** 10.1111/ejn.14726

**Published:** 2020-04-14

**Authors:** Solène Ferreira, Kimberley A. Pitman, Benjamin S. Summers, Shiwei Wang, Kaylene M. Young, Carlie L. Cullen

**Affiliations:** ^1^ Menzies Institute for Medical Research University of Tasmania Hobart Tasmania Australia

**Keywords:** microtubule‐associated protein tau, Myelin, NG2‐glia, oligodendrocyte progenitor cells, tauopathy

## Abstract

Myelin and axon losses are associated with cognitive decline in healthy ageing but are worse in people diagnosed with tauopathy. To determine whether tauopathy is also associated with enhanced myelin plasticity, we evaluated the behaviour of OPCs in mice that expressed a human pathological variant of *microtubule‐associated protein tau* (*MAPT^P301S^*). By 6 months of age (P180), *MAPT^P301S^* mice overexpressed hyperphosphorylated tau and had developed reactive gliosis in the hippocampus but had not developed overt locomotor or memory impairment. By performing cre‐lox lineage tracing of adult OPCs, we determined that the number of newborn oligodendrocytes added to the hippocampus, entorhinal cortex and fimbria was equivalent in control and *MAPT^P301S^* mice prior to P150. However, between P150 and P180, significantly more new oligodendrocytes were added to these regions in the *MAPT^P301S^* mouse brain. This large increase in new oligodendrocyte number was not the result of increased OPC proliferation, nor did it alter oligodendrocyte density in the hippocampus, entorhinal cortex or fimbria, which was equivalent in P180 wild‐type and *MAPT^P301S^* mice. Furthermore, the proportion of hippocampal and fimbria axons with myelin was unaffected by tauopathy. However, the proportion of myelinated axons that were ensheathed by immature myelin internodes was significantly increased in the hippocampus and fimbria of P180 *MAPT^P301S^* mice, when compared with their wild‐type littermates. These data suggest that *MAPT^P301S^* transgenic mice experience significant oligodendrocyte turnover, with newborn oligodendrocytes compensating for myelin loss early in the development of tauopathy.

## Graphical abstract

Mice overexpressing a pathological variant of human microtubule‐associated protein tau (*MAPT* ^P301S^) experience increased oligodendrogenesis in the hippocampus, entorhinal cortex and fimbria between 5 and 6 months of age. Total oligodendrocyte and oligodendrocyte progenitor cell number, axon density and the per cent of myelinated axons remain normal, though there is an increase in newly generated myelin, suggesting that oligodendrocyte turnover occurs early in the development of tauopathy.

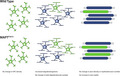

**Abbreviations**
ANOVA: Analysis of variance
ASPA: Aspartoacylase
BCAS1: Breast Carcinoma Amplified Sequence 1
CA1: Cornus Ammonis area 1 of the hippocampus
DNA: Deoxyribonucleaic acid
EdU: 5‐Ethynyl‐2′‐deoxyuridine
FCS: Foetal calf serum
GFP: Green fluorescent protein
HRP: Horseradish peroxidase
MAPT: Microtubule‐associated protein tau
MAPT^P301S^
: T34 isoform of human MAPT (1N4R) with the P301S mutation
OLIG2: Oligodendrocyte transcription factor 2
OPC: Oligodendrocyte progenitor cell
PBS: Phosphate‐buffered saline
PCR: Polymerase chain reaction
PDGFRα: Platelet derive growth factor receptor alpha
*Prnp*
: Prion protein promotor
PVDF: Polyvinylidene difluoride
*SD*
: Standard deviation
*SEM*
: Standard error of the mean
TBS‐T: Tris‐buffered saline with tween‐20
WT: Wild type
YFP: Yellow fluorescent protein

## 1 INTRODUCTION

In healthy ageing, white matter damage can be detected in brain regions that are critical for cognitive and emotional processing, including the hippocampus, neocortex and frontal white matter tracts, and the extent of white matter damage closely correlates with cognitive decline (Charlton et al., 2006; Fan et al., 2019; Hirsiger et al., 2017). However, white matter degeneration is exacerbated in people diagnosed with a tauopathy. In people with frontotemporal dementia, fractional anisotropy, as measured by diffusion tensor imaging, is reduced in frontal and temporal white matter regions, including the anterior corpus callosum, anterior cingulum tracts and uncinate tracts, when compared with healthy controls (Kassubek et al., 2018; Lu et al., 2014; Zhang et al., 2009). Similar studies show that people with Alzheimer's disease have reduced fractional anisotropy in parietal, temporal and frontal regions including the corpus callosum, cingulum and uncinate tracts, compared with controls (Benitez et al., 2014; Brueggen et al., 2019; Choi, Lim, Monteiro, & Reisberg, 2005; O’Dwyer et al., 2011; Stricker et al., 2009; Zhang et al., 2009). In these tauopathies, the observed white matter degeneration likely reflects a combination of myelin breakdown and axon degeneration.

Tauopathies are a group of diseases characterised by the aggregation of hyperphosphorylated tau in neurons and glial cells, including myelinating oligodendrocytes (reviewed by Ferrer, 2018). Tau aggregates in cells of the oligodendrocyte lineage are referred to as coiled bodies and threads and have been identified in post‐mortem tissue from people diagnosed with Pick's disease (Arai et al., 2001; Komori, 1999; Mimuro et al., 2010), progressive supranuclear palsy (Arai et al., 2001; Arima et al., 1997; Jin et al., 2006; Komori, 1999; Nishimura, Ikeda, et al., 1995), corticobasal degeneration (Arai et al., 2001; Feany & Dickson, 1995; Komori, 1999; Wakabayashi et al., 1994), frontotemporal lobar degeneration associated with variants in *microtubule‐associated protein tau* (*MAPT)* (Higuchi et al., 2005) and Alzheimer's disease (Nishimura, Tomimoto, et al., 1995). Frontotemporal dementia is also associated with myelin degeneration in the frontal white matter, and Alzheimer's disease results in an early and progressive reduction in white matter cholesterol and myelin proteins, such as myelin basic protein, proteolipid protein and 2′,3′‐cyclic nucleotide 3′‐phosphodiesterase (CNP) (Roher et al., 2002), and a reduction in CNP expression (Vlkolinský et al., 2001) and impaired myelin lipid synthesis (Couttas et al., 2016) in the frontal grey matter. Myelin degeneration is also detected post‐mortem in the frontal and periventricular white matter regions of people with Alzheimer's disease (Ihara et al., 2010; Zhan et al., 2014), and a recent proteomics study revealed that myelin sheath components are significantly reduced in the frontal cortex of people with sporadic Alzheimer's disease (Zhang et al., 2018).

Tauopathy‐like oligodendrocyte pathology can be induced in mice by the injection of brain tissue homogenates from people who had sporadic Alzheimer's disease, primary age‐related tauopathy, ageing‐related tau astrogliopathy, globular glial tauopathy, progressive supranuclear palsy, Pick's disease or frontotemporal lobar degeneration (linked to the *MAPT^P301L^* variant), into the corpus callosum. Phospho‐tau deposits developed inside cells of the oligodendrocyte lineage and myelin disruption was evident within 6 months of injection (Ferrer et al., 2019). However, a separate study found that tau‐inclusions were rarely seen in mice inoculated with Alzheimer's disease homogenates but were a common feature following inoculation with corticobasal degeneration homogenates (Boluda et al., 2015).

Transgenic mice that express human tauopathy‐associated variants in *MAPT*, primarily the *MAPT^P301L^* and *MAPT^P301S^* variants, also recapitulate many of the aspects of human tauopathy, including the development of gliosis, the formation of neurofibrillary tangles, neuron loss, and motor and cognitive impairment (Lewis et al., 2000; Lin, Lewis, Yen, Hutton, & Dickson, 2003a, 2003b; Ramsden et al., 2005; Ren et al., 2014; Santacruz et al., 2005; Takeuchi et al., 2011; Yoshiyama et al., 2007). In the spinal cord of the *Prnp‐MAPT^P301L^* transgenic mice, oligodendrocytes also undergo apoptosis (Zehr et al., 2004). Furthermore, when three human tauopathy *MAPT* variants are expressed under the control of the mouse α‐tubulin promoter, in the absence of endogenous *Mapt*, coiled bodies form inside spinal cord oligodendrocytes, and oligodendrocyte number is reduced by 6 months of age—prior to neuron loss (Higuchi et al., 2002). Consistent with these findings, the expression of *MAPT^P301L^* in *CamKIIa* ^+^ neurons was associated with thinner myelin ensheathing perforant pathway axons that project from the entorhinal cortex to the hippocampus (Jackson et al., 2018), and when expression of this variant was restricted to oligodendrocytes (*CNP* promoter), myelin degeneration and axon loss from the spinal cord were detected prior to the development of tau aggregates in oligodendrocytes or oligodendrocyte loss (Higuchi et al., 2005).

It is possible that oligodendrogenesis occurs alongside oligodendrocyte loss in tauopathy, as oligodendrocyte progenitor cells (OPCs) have the ability to proliferate and differentiate to produce new oligodendrocytes in response to oligodendrocyte loss and demyelination (Assinck et al., 2017; Baxi et al., 2017; Tripathi et al., 2010; Zawadzka et al., 2010). This has not been examined in detail in preclinical models of tauopathy; however, young adult *Thy1.2‐MAPT^P301S^* mice may remyelinate more effectively than controls. Following a focal injection of lysolecithin into the spinal cord ventral funiculus, OPC density was maintained, but the density of oligodendrocytes and the expression of myelin basic protein were elevated at the lesion site of *Thy1.2‐MAPT^P301S^* mice compared with demyelinated wild‐type controls (Ossola et al., 2016). In this study, we demonstrate that the overexpression of *MAPT^P301S^*, primarily in neurons, results in a large number of new oligodendrocytes accumulating in the hippocampus, entorhinal cortex and fimbria between 5 and 6 months of age, and an increase in the number of axons ensheathed by immature myelin internodes. As this increase in oligodendrocyte addition occurred prior to axon loss or the development of overt cognitive deficits and did not increase oligodendrocyte density or the proportion of axons that were myelinated in these regions, it is likely that new oligodendrocyte addition facilitates oligodendrocyte and myelin maintenance as early pathology develops in the central nervous system (CNS) of *MAPT^P301S^* transgenic mice.

## 2 MATERIALS AND METHODS

### 2.1 Animals

*Rosa26‐YFP* cre‐sensitive reporter mice (Srinivas et al., 2001) were purchased from the Jackson Laboratory [B6.129X1‐Gt(ROSA)26Sortm1(EYFP)Cos/J, stock #006148] and backcrossed onto a C57BL/6 background in house for >10 generations. *Prnp‐MAPT^P301S^* (*MAPT^P301S^*) transgenic mice (Yoshiyama et al., 2007), that express a human variant of *MAPT,* were purchased from the Jackson Laboratory [B6;C3‐Tg(Prnp‐MAPT*P301S)PS19Vle/J, stock #008169] and backcrossed onto a C57BL/6 background for >20 generations. *Pdgfrα‐CreER^T2^* transgenic mice (Rivers et al., 2008) were a kind gift from Prof. William D Richardson (University College London, UK). Mice were maintained on a C57BL/6 background and bred to generate experimental mice that were heterozygous for each transgene. Male and female mice were housed in individually ventilated cages (Optimice) on a 12‐hr light/dark cycle (07:00–19:00), with food and water available ad libitum. All animal experiments were approved by the Animal Ethics Committee of the University of Tasmania (13,741 and 16,151) and carried out in accordance with the Australian code of practice for the care and use of animals in science. Details of animal experiments are reported in accordance with the ARRIVE guidelines.

### 2.2 Genotyping

Genomic DNA extractions and the detection of the *Cre recombinase* and the *Rosa26‐YFP* transgenes by polymerase chain reaction (PCR) were carried out as previously described (O’Rourke et al., 2016). Each PCR to detect the *MAPT* transgene was carried out using Taq DNA polymerase with standard Magnesium‐free Taq buffer (M0329L; New England BioLabs) and deoxynucleotide (dNTP) solution mix (N0447L; New England BioLabs), with the following primers: MAPT 5′ GGG GAC ACG TCT CCA CGG CAT CTC AGC AAT GTC TCC and MAPT 3′ TCC CCC AGC CTA GAC CAC GAG AAT, and was heated to 94°C for 4 min and amplified across 35 cycles of 94°C for 30s, 57°C for 45s and 72°C for 60s, followed by a final 10 min at 72°C, to yield a DNA fragment of ~350 bp. DNA products were run on a 2% (w/v) agarose gel in TAE containing SYBR safe (Thermo Fisher Scientific) and visualised using an Amersham Imager 600 (Ge Healthcare Life Sciences, UK).

### 2.3 Tamoxifen preparation and delivery

Tamoxifen (Sigma; cat # T5648) was dissolved in corn oil (40 mg/ml) by sonication (Ultrasonic cleaner FXP 8M, Unisonics Australia) at 21°C for 2 hr. For lineage tracing, adult mice (P60) received 300 mg tamoxifen/kg body weight daily for four consecutive days by oral gavage as previously described (O’Rourke et al., 2016). Mice were analysed 7, 90 or 120 days after their first dose of tamoxifen and are referred to as P60 + 7, P60 + 90 and P60 + 120, respectively.

### 2.4 Western blotting

Mice were terminally anaesthetised using sodium pentobarbital (i.p 100 mg/kg) and transcardially perfused with ice‐cold 0.01 M phosphate‐buffered saline (PBS; *n* = 3 mice per group). On ice, the dorsal region of the hippocampus was collected from 2mm thick coronal slices spanning Bregma −1.06 to −2.06 (Franklin & Paxinos, 2007), and then prepared and analysed by Western blot as per Auderset, Cullen, and Young (2016). Briefly, precast Bolt™ 4%–12% Bis‐Tris Plus Gels (Life Technologies, Australia) were run for 1 hr at 21°C and 90v, followed by 20 min at 165v. The proteins were transferred onto an ethanol‐activated PVDF membrane (BioRad) over a 60‐min period at 20v and 4°C. The membrane was blocked for 1 hr at 21°C by immersion in 0.2% (v/v) Tween‐20 in Tris‐buffered saline (TBS‐T) containing 5% (w/v) skim milk powder, before being transferred to TBS‐T containing 5% (w/v) skim milk powder and either rabbit anti‐E178 (1:1,000, Abcam; detects human and mouse tau) or rabbit anti‐Thr231 (1:1,000, Abcam; detects human and mouse phosphorylated tau) overnight at 4°C. Each membrane was washed thrice in TBS‐T before being incubated for 1 hr at 21°C in TBS‐T containing 1% (w/v) skim milk powder and goat anti‐rabbit horseradish peroxidase (HRP)‐conjugated secondary antibody (1:10,000, Dako). Each membrane was washed in TBS‐T before being exposed to a 1:1 mix of Immobilon Western™ HRP Peroxidase Solution (Millipore) and Luminol Reagent (Millipore), to visualise the protein bands on an Amersham Imager 600 (Ge Healthcare Life Sciences, UK). To control for protein loading, membranes were washed with PBS, TBS‐T and blot stripping buffer (ThermoScientific), incubated for 1h at 21°C with mouse anti‐β‐actin (1:1,000, Sigma), washed thrice in TBS‐T and incubated for 1 hr at 21°C with secondary goat anti‐mouse HRP (1:10,000, Dako). Western blot band intensity was calculated by measuring integrated density and normalising this signal to β‐actin protein expression levels.

### 2.5 Locomotor and cognitive testing

Behavioural testing was carried out for *MAPT^301S^* transgenic mice, their wild‐type (WT) littermates and C57BL/6 mice in separate cohorts at 60 (*n* = 23 WT, 9 *MAPT^P301S^*), 90 (*n* = 24 WT, 12 *MAPT^P301S^*) and 180 (*n* = 24 WT, 13 *MAPT^P301S^*) days of age. All behavioural testing was carried out during the dark phase of the light–dark cycle. Mice were moved to the testing room 2 hr prior to the light cycle change and habituated to the room for 3 hr. All testing was carried out within the same 5‐hr window of the dark phase. Sodium lights were used in the room, and bright lights were used above the maze as needed. All trials were video recorded and animal movement tracked using automated tracking software (EthoVision XT 11, Noldus, Netherlands). Males were tested prior to females, but the order of testing was otherwise randomised between sessions. All equipment was cleaned with 70% ethanol between trials.

T‐Maze: The T‐maze was performed using a protocol adapted from Deacon and Rawlins (2006). A mouse was placed in the start arm, and once they chose to explore the left or right arm of the maze, retreat from that arm was blocked for 1 min. The mouse was then returned to the start arm and allowed to make another choice. This was repeated 10 times. Mice naturally exhibit exploratory behaviour and tend to choose the arm not visited in the previous trial; therefore, returning to the same arm in successive trials was recorded as an error.

Open field and novel object recognition: On day 1, we carried out an open field assessment using a protocol adapted from Wang et al. (2013), to assess locomotor and anxiety‐like behaviour. Each mouse was placed in an open square arena (30 cm^2^, with walls of 20 cm in height) lit (200 lux) to create a bright centre and dark perimeter, and the speed of movement and total distance moved was measured over a 10 min period. On day 2, the arena was uniformly illuminated (50 lux) and contained two identical objects (multi‐coloured green and blue Lego towers), equidistant from the box edges (7.5 cm away from the box edges). Each mouse was left to explore the arena and familiarise themselves with the identical objects for 10 min. On day 3, one of the familiarised objects was replaced by a novel object (a multi‐coloured green and blue Lego man) that was of similar size and colour, but a different shape and texture. Each mouse was returned to the arena and left to explore for 5 min. The time spent exploring each object was recorded, and the proportion of time exploring the novel object was calculated as an indication of short‐term recognition memory.

Barnes Maze: Mice underwent a shortened version of the Barnes maze protocol, adapted from Attar et al. (2013). On day 1, mice were placed in the brightly lit centre (120 lux) of an elevated (30 cm above the ground), circular maze (100 cm diameter) that contained 20 holes evenly spaced around the circumference. After 1 min, the mice were gently directed to an escape box located underneath one of the holes in the circumference and left to habituate to the box for 5 min. On days 2 and 3, the maze was raised to 70 cm, and light intensity in the centre of the maze increased to 160 lux. Distinct patterns were placed on each wall surrounding the maze, acting as spatial reference points that remained consistent throughout all trials. At the start of each trial, each mouse was placed at the centre of the maze under a covered start box for 15–30 s before the box was removed, and the mouse left to explore until they found the escape box or 5 min elapsed. If a mouse did not find the escape box prior to the end of trial, it was given direction to the box and allowed to enter it. After entering the escape box, each mouse was left for 1 min before being returned to the home cage to await the next trial. Mice were trained to learn the location of the escape box across 3 trials per day with an inter‐trial interval of 30–45 min. During training, approaching any hole that did not lead to the escape box was considered a primary error, and the number of primary errors made during a trial was measured as an indicator of learning (reviewed by Gawel, Gibula, Marszalek‐Grabska, Filarowska, & Kotlinska, 2019).

Short‐term memory and long‐term memory were assessed 1 day and 2 weeks after initial training, respectively. For each memory probe trial, mice were returned to the maze, with the escape box now removed, and were left to explore the maze for 5 min. The maze was divided into four quadrants within the tracking software (EthoVision XT 11) and the quadrant containing the hole that previously led to the escape hole was designated the target zone. The proportion of time spent within the target zone during the probe trial was measured as an indicator of intact memory for the location of the escape box.

### 2.6 Immunohistochemistry

Tissue fixation and cryoprotection were performed as previously described (O’Rourke et al., 2016). About 30 µm coronal brain cryosections containing the hippocampus, entorhinal cortex and fimbria (Bregma −1.34 to −2.7; Franklin & Paxinos, 2007) were collected and processed as floating sections. Cryosections were incubated for 1 hr at 21°C in blocking solution [10% foetal calf serum (FCS)/ 0.1% triton x‐100 in PBS] before being placed on an orbital shaker overnight at 4°C in blocking solution containing primary antibodies [goat anti‐PDGFRα (1:100, R&D Systems); rat anti‐GFP (1:2000, Nacalai Tesque); rabbit anti‐OLIG2 (1:400, Merck Millipore); rabbit anti‐ASPA (1:200, Merck Millipore); rabbit anti‐Ki67 (1:200, Abcam); mouse anti‐NaBC1 (BCAS1; 1:200, Santa Cruz) or guinea pig anti‐Iba1 (1:250, Synaptic Systems)]. Sections were washed thrice in PBS before being placed on an orbital shaker at 4°C overnight, in blocking solution containing secondary antibodies, conjugated to Alexa Fluors [Life Technologies Corporation: donkey anti‐rat 488 (1:500); donkey anti‐goat 568 (1:1,000); donkey anti‐rabbit 647 (1:1,000), donkey anti‐goat 647 (1:1,000) or goat anti‐guinea pig 488 (1:1,000)]. Cell nuclei were visualised by the inclusion of Hoechst 33342 (1:10,000, Invitrogen). Floating sections were mounted onto glass slides and the fluorescence preserved by the application of fluorescent mounting medium (Dako Australia Pty. Ltd., Campbellfield, Australia).

### 2.7 EdU administration and detection

5‐Ethynyl‐2′‐deoxyuridine (EdU; Invitrogen) was administered to P175 mice (*n* = 6 WT, *n* = 3 *MAPT^P301S^*) via their drinking water (0.2 mg/ml, as per Young et al., 2013) for five consecutive days. EdU‐labelled cells were visualised using the AlexaFluor‐647 Click‐iT EdU kit (Invitrogen). Briefly, 30 µm floating cryosections were incubated for 15 min in 0.5% Triton X‐100 (v/v) in PBS at room temperature before being transferred into the EdU developing cocktail and incubated for 45 min in the dark. Cryosections were washed twice in PBS before carrying out immunohistochemistry as described above.

### 2.8 Confocal microscopy and cell quantification

Confocal images were collected using an Andor spinning disk confocal microscope with NIS Elements Software (Andor Technology Ltd., Belfast, Northern Ireland) or an UltraView Nikon Ti spinning disk confocal microscope with Volocity software (Perkin Elmer, Waltham, USA). For quantification of cell density, low magnification images were taken of the hippocampus, entorhinal cortex and fimbria, using 20× air objective. Multiple z stack images (3 µm z‐spacing) were collected using standard excitation and emission filters for DAPI, FITC (Alexa Fluor‐488), TRITC (Alexa Fluor 568) and CY5 (Alexa Fluor 647) and stitched together to make a composite image of the entire region of interest. Each region of interest was defined based on Hoechst nuclear staining and according to the Mouse Brain Atlas (Franklin & Paxinos, 2007) and *n* ≥ 3 brain sections were imaged per mouse. Cell quantification was performed manually using Fiji software (NIH, Washington DC, USA) or Adobe Photoshop. For cell density calculations, the total number of cells counted within a region of interest was divided by the total x–y area and expressed as cells per mm^2^ (note that z‐tissue depth was always 30 µm). All quantification from confocal images was carried out by an experimenter blind to age and genotype for *n* = 3–6 mice per group and was additionally validated by a second blinded experimenter.

### 2.9 Transmission electron microscopy

Mice were terminally anaesthetised using sodium pentobarbital (i.p 100 mg/kg) and transcardially perfused with Karnovsky's fixative [0.8% (v/v) glutaraldehyde/2% (w/v) PFA/0.25 mM CaCl_2_/0.5 mM MgCl_2_ in 0.1 M sodium cacodylate buffer]. Brains were sliced into 2 mm thick coronal slices using a rodent brain matrix (Agar Scientific, Essex, UK) and immersion fixed at 21°C for 2 hr, before being stored in 0.1 M sodium cacodylate buffer overnight at 4°C. 1mm sections of the stratum lacunosum moleculare of the Cornu Ammonis subfield 1 (CA1) of the hippocampus or the fimbria were dissected and incubated in 1% osmium tetroxide/1.5% potassium ferricyanide in 0.065 M sodium cacodylate buffer, in the dark, for 2 hr at 4°C. Tissue was washed 5 times in Milli‐Q water, before being dehydrated in 70% ethanol (v/v) in Milli‐Q water overnight at 21°C; 80% ethanol (2 × 10 min); 85% ethanol (2 × 10 min); 90% ethanol (2 × 10 min); 95% ethanol (2 × 10 min) and 100% ethanol (4 × 10 min). Tissue was embedded by serial exposure to 100% propylene oxide (2 × 5 min); 75% propylene oxide/25% epon (4 hr); 67% propylene oxide/33% epon (4 hr); 50% propylene oxide/50% epon (overnight); 33% propylene oxide/67% epon (4 hr); 25% propylene oxide/75% epon (4 hr) and 100% epon (overnight). Tissue was transferred to fresh 100% epon for 4h before being polymerised at 60°C for 72 hr.

About 70 nm ultramicrotome (Reichert Ultracut S, Leica) sections were collected using a diamond knife (Diatome) and were floated on Milli‐Q water. Floating sections were collected with a perfect loop (Diatome) and placed on a gold grid with formvar (ProSciTech) and stained with Reynolds’ lead citrate stain [Reynolds, 1963; lead nitrate (Sigma) and trisodium citrate dihydrate (Merck)] and with filtered 4% uranyl acetate (Serva) in 50% ethanol to enhance the contrast. Electron micrographs were collected using a HT7700 (Hitachi) transmission electron microscope. Image analysis was carried out using Fiji software (NIH, Washington DC, USA). Axons were identified based on their microtubule organisation (reviewed by Stassart et al., 2018) and individual myelin lamellae (wraps) by the presence of major dense lines (reviewed by Simons & Nave, 2016). New myelin was defined as myelin that contained <5 wraps as well as a large cytoplasmic layer (inner tongue) between the axon and the myelin (Kang et al., 2013; Liu et al., 2012). The g‐ratio was measured for a minimum of 50 myelinated axons per region per mouse. The number of myelin wraps per sheath was quantified in axons that were ensheathed by mature compact myelin (no enlarged cytoplasmic layer) for a minimum of 30 axons per mouse. Quantification was performed by an experimenter blind to genotype for *n* = 3–4 mice per group.

### 2.10 Statistical analyses

Statistical analyses were performed using GraphPad Prism 8.0 (La Jolla CA, USA). The distribution of each data set was evaluated to determine whether the data were normally distributed using the d’Agostino and Pearson normality test or Shapiro–Wilk normality test as required. Data that were normally distributed were analysed by a parametric test [two‐tailed unpaired *t* test, one‐way analysis of variance (ANOVA) or two‐way ANOVA for group comparisons with a Bonferroni post hoc test], and data that were not normally distributed were analysed using a non‐parametric test [simple linear regression analysis]. A survival curve comparison was performed using a log‐rank (Mantel‐Cox) test. Statistical significance was established as *p* < .05. *p*‐Values for main genotype comparisons are included in text where appropriate, but full statistical details are reported in the corresponding figure legends. Individual data points are presented on each graph, and further data supporting these findings will be made available by the corresponding author upon reasonable request. Behavioural data are presented as mean ± standard error of the mean (*SEM*). Western blot and immunohistochemical data are presented as mean ± standard deviation (*SD*).

## 3 RESULTS

### 3.1 MAPT^P301S^ transgenic mice do not develop overt locomotor or memory impairment by P180

**Figure 1:**
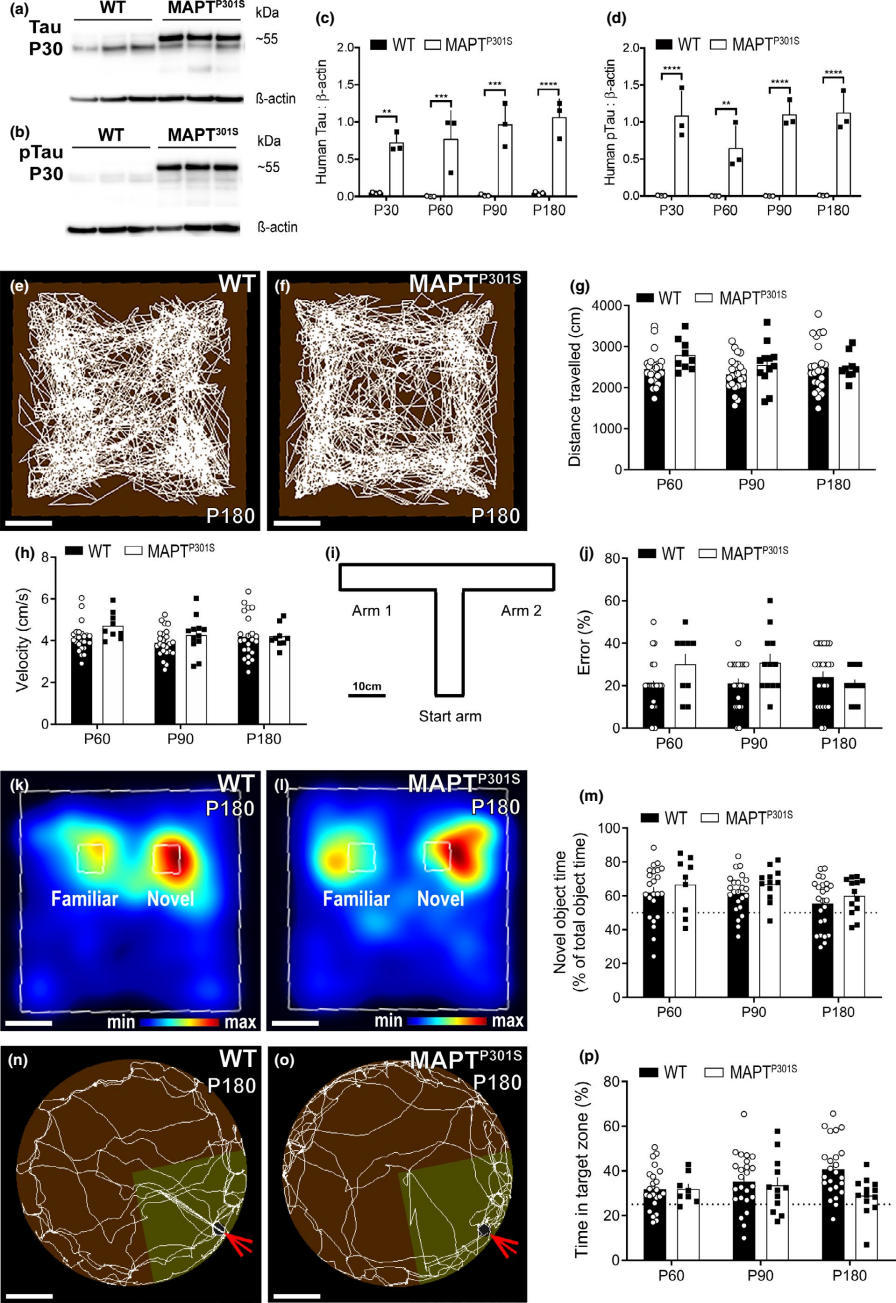
*MAPT^P301S^* mice do not develop overt locomotor or memory impairment by P180 (a‐b) Representative Western blots probing for tau protein (a; human ~55 kDa; mouse ~51 kDa) and phosphorylated Tau (pTau) protein (b; human ~55 kDa, mouse ~51 kDa) in hippocampal brain lysates from P30 wild‐type (WT; open circles, black bars) and *MAPT^P301S^* (black squares, open bars) mice. (c) Quantification of human Tau protein expression relative to β‐actin expression in P30, P60, P90 and P180 WT and *MAPT^P301S^* mice [two‐way ANOVA, genotype: *F* (1, 16) = 110, *p* < .0001; age: *F* (3, 16) = 1.034, *p* = .4040; interaction: *F* (3, 16) = 0.9767, *p* = .4282]. (d) Quantification of human phosphorylated tau expression relative to β‐actin expression *MAPT^P301S^* and WT mice at P30, P60, P90 and P180 [two‐way ANOVA, genotype: *F* (1, 16) = 148.4, *p* < .0001; age: *F* (3, 16) = 2.078, *p* = .1434; interaction: *F* (3, 16) = 2.004, *p* = .1540]. (e‐f) Representative track visualisation images (EthoVision XT) showing movement (white lines) of P180 WT (e) and *MAPT^P301S^* (f) mice during the open field locomotor task. (g) Quantification of the total distance travelled by WT and *MAPT^P301S^* mice in the open field task at P60, P90 and P180 [two‐way ANOVA, genotype: *F* (1, 94) = 3.536, *p* = .0631; age: *F* (2, 94) = 1.180, *p* = .3119; interaction: *F* (2, 94) = 0.767, *p* = .4671]. (h) Quantification of the average movement velocity of WT and *MAPT^P301S^* mice during the open field task at P60, P90 and P180 [two‐way ANOVA, genotype: *F* (1, 94) = 3.466, *p* = .0658; age: *F* (2, 94) = 1.253, *p* = .2904; interaction: *F* (2, 94) = 0.8427, *p* = .4338]. (i) Schematic of the T‐maze apparatus. (j) Quantification of the proportion incorrect arm choices (errors) made by P60, P90 and P180 WT and *MAPT^P301S^* mice during the T‐maze alternation task [two‐way ANOVA, genotype: *F* (1, 96) = 4.317, *p* = .0404; age: *F* (2, 96) = 0.7271, *p* = .4859; interaction: *F* (2, 96) = 3.028, *p* = .0531]. (k‐l) Representative heat maps (EthoVision XT) showing the relative proportion of time P180 WT (k) and MAPT (l) mice spent exploring the familiar and novel objects during the novel object recognition task. Warmer colours represent a greater proportion of time in that area. (m) Quantification of the proportion of time P60, P90, and P180 WT or *MAPT^P301S^* mice spent exploring the novel object relative to the total time spent exploring either object [two‐way ANOVA, genotype: *F* (1, 98) = 2.823, *p* = .0961; age: *F* (2, 98) = 2.506, *p* = .0868; interaction: *F* (2, 98) = 0.006544, *p* = .9935]. (n‐o) Representative track visualisation images (EthoVision XT) showing movement (white lines) of P180 WT (n) and *MAPT^P301S^* (o) mice during the Barnes maze long‐term memory probe trial, carried out 2 weeks after mice learned the expected location of an escape box (red arrows). Yellow shading indicates the quadrant of the maze defined as the target zone. (p) Quantification of the proportion of time P60, P90, and P180 WT or *MAPT^P301S^* mice spent within the target zone during the long‐term memory probe trial [two‐way ANOVA, genotype: *F* (1, 98) = 3.708, *p* = .0570; age: *F* (2, 98) = 0.5633, *p* = .5711; interaction: *F* (2, 98) = 2.623, *p* = .0777]. Western blot data are presented as mean ± *SD*, *n* = 3 mice per group. Behaviour data are presented as mean ± *SEM*, *n* = 9–24 mice per group. Asterisks denote significant differences identified by Bonferroni post hoc analysis, **p < .01, ***p < .001, ****p < .0001. Scale bars represent 10 cm (e‐f, i, k‐l) and 25 cm (n‐o)

Prior to examining the response of cells of the oligodendrocyte lineage to the earliest stages of tauopathy, we confirmed that human tau was expressed in brain tissue from *MAPT^P301S^* transgenic mice (Figure 1). Western blot analysis of the dorsal hippocampus indicated that human tau (Figure 1a,c) and phosphorylated human tau (Figure 1b,d) were expressed by *MAPT^P301S^* mice, but not their wild‐type (WT) littermates, at P30, P60, P90 and P180. By comparing the relative expression of human tau (upper band, Figure 1a) and endogenous mouse tau (lower band, Figure 1a), we determined that human tau expression was twofold to fivefold more abundant than mouse tau in the hippocampus of *MAPT^P301S^* mice (Fig. S1a). We also found that phosphorylated human tau (upper band, Figure 1b) was sevenfold to 10‐fold more abundant than phosphorylated mouse tau (lower band, Figure 1b) in the hippocampus of *MAPT^P301S^* mice (Fig. S1b). This transgenic overexpression of human tau was associated with the impaired survival of *MAPT^P301S^* transgenic mice, relative to their WT littermates, when they were followed until P200 (Fig. S1c, *p* = .04), as well as a visible increase in reactive microgliosis in the hippocampus and entorhinal cortex, and to a lesser extent the fimbria at P180 (Fig. S2).

MAPT^P301S^ mice typically do not develop locomotor or cognitive impairments until after 10 months of age (Chalermpalanupap et al., 2017; Dumont et al., 2011). Before characterising the behaviour of oligodendrocyte lineage cells in the pre‐symptomatic stage of disease, we first confirmed that the overexpression of human tau did not induce locomotor or cognitive deficits before 6 months of age by subjecting WT and MAPT^P301S^ mice to a battery of behavioural tasks. WT (Figure 1e) and *MAPT^P301S^* (Figure 1f) mice were first placed in an open field arena, and the distance that each mouse travelled (Figure 1g), and the velocity of that movement (Figure 1h), was mapped over a 10‐min period. The overexpression of human tau did not alter the distance travelled (Figure 1g, *p* = .06) or the velocity of movement (Figure 1h, *p* = .06) at P60, P90 or P180. WT and *MAPT^P301S^* mice also spent a similar proportion of time in the central area of the open field [P60: WT 31.9 ± 2.0%, *MAPT^P301S^* 38.2 ± 3.8%; P90: WT 32.2 ± 2.0%, *MAPT^P301S^* 32.3 ± 3.5%; P180: WT 35.8 ± 1.9%, *MAPT^P301S^* 35.1 ± 3.3%, mean ± *SEM*; two‐way ANOVA, genotype: *F* (1, 94) = 0.6999, *p* = .40; age: *F* (2, 94) = 0.8799, *p* = .41; interaction: *F* (2, 94) = 0.9449, *p* = .39].

Working memory was evaluated by measuring spontaneous alternation in the T‐maze (Figure 1i). We found that WT and *MAPT^P301S^* mice performed similarly in this task, with mice of each genotype making an equivalent number of errors at P60 (*p* = .10), P90 (*p* = .09) and P180 (*p* > .99; Figure 1j). Short‐term recognition memory was evaluated for WT (Figure 1k) and *MAPT^P301S^* (Figure 1l) mice using the novel object recognition task. Both WT and *MAPT^P301S^* mice spent a larger proportion of their time exploring the novel object, compared with the familiar object, and we found that the overexpression of human tau did not affect the ability of mice to discriminate between the objects at P60, P90 or P180 (Figure 1m, *p* = .09).

The spatial learning ability, as well as the short‐ and long‐term memory performance of WT (Figure 1n) and *MAPT^P301S^* (Figure 1o) mice, was assessed using a Barnes maze spatial learning task (Fig. S3a). We found that at P60, P90 and P180, WT and *MAPT^P301S^* mice made fewer visits to incorrect holes (primary errors) on day 2 of training, compared with day 1 (P60: *p* = .0008, P90: *p* = .0005 and P180: *p* = .02), suggesting that mice of both genotypes learned the location of the escape box (Fig. S3b‐c). One day after training, during the short‐term memory probe phase, P180 WT and *MAPT^P301S^* mice spent an equivalent amount of time in the target quadrant of the maze (*p* = .40), and both groups spent more time in the target quadrant compared with all other quadrants (Fig. S3d). During the long‐term memory probe phase, two weeks after initial training, WT mice spent more time in the target quadrant, relative to other maze quadrants, while *MAPT^P301S^* mice spent an equivalent amount of time in all 4 quadrants (Fig. S3e). While these data may suggest that *MAPT^P301S^* mice are beginning to experience long‐term memory impairment, this was not a robust phenotype, as the time that *MAPT^P301S^* mice spent in the target quadrant was equivalent to that of WT mice (Figure 1p; *p* = .06). Overall, these data indicate that human tau overexpression does not induce overt cognitive impairment in mice by P180.

### 3.2 The number of new YFP^+^ cells produced by OPCs is elevated in P180 MAPT^P301S^ mice

**Figure 2:**
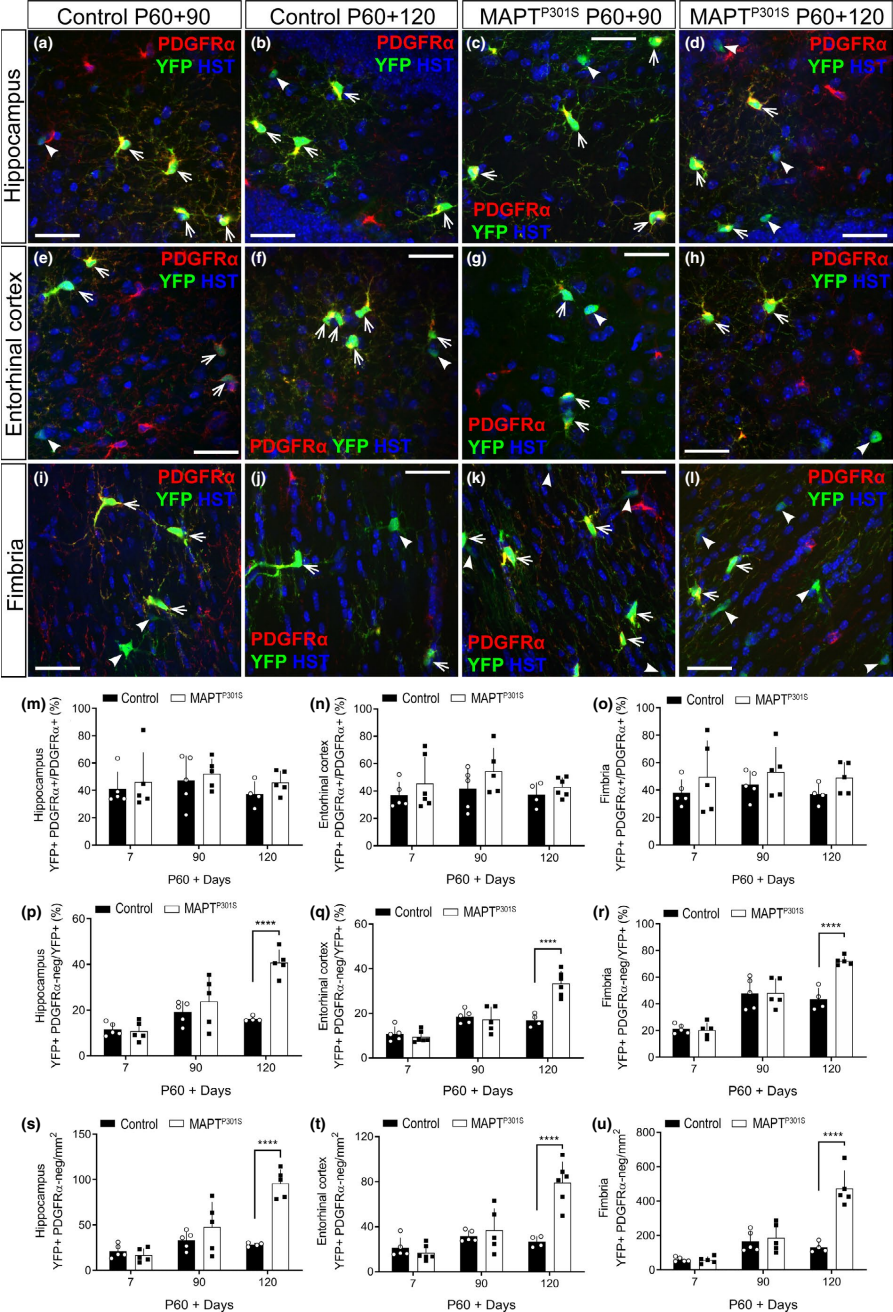
New oligodendrocyte addition is increased in the hippocampus, entorhinal cortex and fimbria of *MAPT^P301S^* mice at P180. (a‐l) Representative confocal images showing PDGFRα (red), YFP (green) and Hoechst (blue) in the hippocampus (a‐d), entorhinal cortex (e‐h) and fimbria (i‐l) of *Pdgfrα‐CreER^T2^::Rosa26‐YFP* (control; a‐b, e‐f, i‐j) and *Pdgfrα‐CreER^T2^::Rosa26‐YFP::Prnp‐MAPT^P301S^* (*MAPT^P301S^*; c‐d, g‐h, k‐l) mice at 90 (P60 + 90) and 120 (P60 + 120) days post tamoxifen administration (P60). (m‐o) Quantification of the proportion of recombined OPC (YFP^+^ PDGFRα^+^/PDGFRα^+^) in the hippocampus [(m): two‐way ANOVA, genotype: *F* (1, 23) = 1.272, *p* = .2710; age: *F* (2, 23) = 0.8038, *p* = .4598; interaction: *F* (2, 23) = 0.04821, *p* = .9530], the entorhinal cortex [(n): two‐way ANOVA, genotype: *F* (1, 25) = 3.13, *p* = .0891; age: *F* (2, 25) = 0.982, *p* = .3885; interaction: *F* (2, 25) = 0.169, *p* = .8455] and the fimbria [(o): two‐way ANOVA, genotype: *F* (1, 23) = 3.469, *p* = .0753; age: *F* (2, 23) = 0.3587, *p* = .7024; interaction: *F* (2, 23) = 0.02172, *p* = .9785] of control (open circles, black bars) and *MAPT^P301S^* (black squares, open bars) mice at 7, 90 and 120 days post tamoxifen administration (P60). (p‐r) Quantification of the proportion of recombined OPCs that differentiated into new oligodendrocytes (PDGFRα‐neg YFP^+^/YFP^+^) over 7, 90 and 120 days in the hippocampus [(p): two‐way ANOVA, genotype: *F* (1, 23) = 18.33, *p* = .0003; age: *F* (2, 23) = 20.33, *p* < .0001; interaction: *F* (2, 23) = 11.70, *p* = .0003], the entorhinal cortex [(q): two‐way ANOVA, genotype: *F* (1, 25) = 9.489, *p* = .0050; age: *F* (2, 25) = 34.13, *p* < .0001; interaction: *F* (2, 25) = 15.42, *p* < .0001] and the fimbria [(r): two‐way ANOVA, genotype: *F* (1, 23) = 10.18, *p* = .0041; age: *F* (2, 23) = 58.20, *p* < .0001; interaction: *F* (2, 23) = 10.49, *p* = .0006] of control and *MAPT^P301S^* mice. (s‐u) Quantification of the density of new oligodendrocytes (PDGFRα‐neg YFP^+^/mm^2^) added to the hippocampus [(s): two‐way ANOVA, genotype: *F* (1, 23) = 23.09, *p* < .0001; age: *F* (2, 23) = 20.88, *p* < .0001; interaction: *F* (2, 23) = 15.26, *p* < .0001], the entorhinal cortex [(t): two‐way ANOVA, genotype: *F* (1, 25) = 14.94, *p* = .0007; age: *F* (2, 25) = 18.28, *p* < .0001; interaction: *F* (2, 25) = 14.37, *p* < .0001] and the fimbria [(u): two‐way ANOVA, genotype: *F* (1, 23) = 26.15, *p* < .0001; age: *F* (2, 23) = 35.02, *p* < .0001; interaction: *F* (2, 23) = 21.65, *p* < .0001]. Data are presented as mean ± *SD*, *n* = 3–13 mice per group. Asterisks indicate significant differences identified by Bonferroni post hoc analysis, ****p < .0001. Scale bars represent 30 µm. Arrows indicate YFP^+^ PDGFRα^+^ recombined OPCs. Arrow heads indicate YFP^+^ PDGFRα‐neg newly added oligodendrocytes

To determine whether the overexpression of human hyperphosphorylated tau could influence adult oligodendrogenesis, prior to the onset of a behavioural change, we performed cre‐lox lineage tracing of PDGFRα^+^ OPCs in the hippocampus, entorhinal cortex and fimbria of control (*Pdgfrα‐CreER^T2^::Rosa26‐YFP*) and *MAPT^P301S^* (*Pdgfrα‐CreER^T2^::Rosa26‐YFP::Prnp*‐*MAPT^P301S^*) mice. The hippocampus and entorhinal cortex were selected, as they are among the first regions affected in human tauopathy (Du et al., 2001; Pennanen et al., 2004; Xu et al., 2000), and the fimbria is part of the major white matter tract that connects the hippocampi of both hemispheres and each hippocampus with other subcortical structures of the brain (Fimbria–fornix commissural pathway; Kesner & Rolls, 2015; Wyss et al., 1980). Tamoxifen was administered to P60 control and *MAPT^P301S^* mice, and brain tissue was collected at P60 + 7, 90 or 120 days (Figure 2). Coronal cryosections containing the hippocampus (Figure 2a‐d, Fig. S4a‐h), entorhinal cortex (Figure 2e‐h) and fimbria (Figure 2i‐l, Fig. S4i‐n) were stained to detect of PDGFRα^+^ (red), yellow fluorescent protein (YFP; green), OLIG2 and Hoechst 33342 (blue). By quantifying the proportion of OPCs that expressed YFP, we determined that ~40% of OPC had undergone tamoxifen‐induced recombination in all brain regions examined in control and *MAPT^P301S^* mice (Figure 2m‐o) and these YFP‐labelled OPCs gave rise to YFP^+^ PDGFRα‐negative cells over time (arrows, Figure 2a‐l). Between P60 + 90 and P60 + 120, the proportion of YFP^+^ cells that were PDGFRα‐negative significantly increased in the hippocampus (Figure 2p, *p* = .0005), entorhinal cortex (Figure 2q, *p* < .0001) and fimbria (Figure 2r **,** *p* = .0002) of *MAPT^P301S^* mice, despite being largely unchanged in controls. These YFP^+^ PDGFRα‐negative cells were new oligodendrocytes, as they labelled for OLIG2. Indeed, 97.9% ± 1.4% of all YFP^+^ cells in the hippocampus of P60 + 120 control and 96.2% ± 1.3% of all YFP^+^ cells in the hippocampus of P60 + 120 *MAPT^P301S^* transgenic mice co‐labelled with OLIG2^+^, confirming that they remain within the oligodendrocyte lineage (mean ± *SD*, unpaired *t* test: *t* = 1.17, *df* = 4, *p* = .30; *n* = 3 mice per genotype; Fig. S4). Therefore, the density of new YFP^+^ oligodendrocytes present in each brain region essentially doubled over a one‐month period in *MAPT^P301S^* mice, such that by P60 + 120 *MAPT^P301S^* mice had significantly more new oligodendrocytes in the hippocampus (Figure 2s, *p* < .0001), entorhinal cortex (Figure 2t, *p* < .0001) and fimbria (Figure 2u, *p* < .0001), than control mice.

### 3.3 Fimbria OPC proliferation is increased in P180 MAPT^P301S^ mice

**Figure 3:**
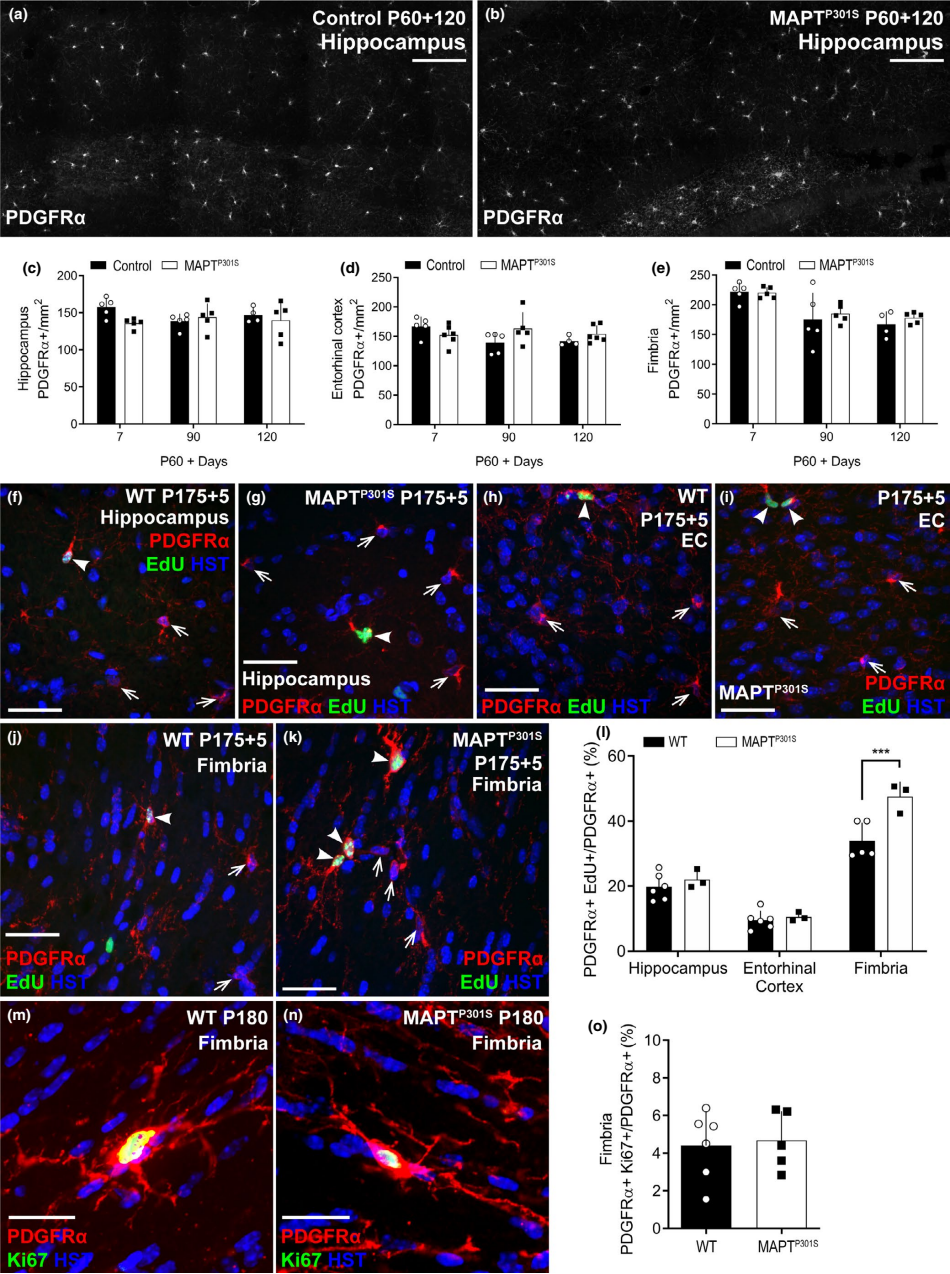
OPC proliferation is increased in the fimbria of *MAPT^P301S^* mice at P180. (a‐b) Representative confocal image stacks showing Pdgfrα labelling in the hippocampus of *Pdgfrα‐CreER^T2^::Rosa26‐YFP* (control; a) and *Pdgfrα‐CreER^T2^::Rosa26‐YFP::Prnp‐MAPT^P301S^* (*MAPT^P301S^*; b) mice at 120 days post tamoxifen administration (P60). (c‐e) Quantification of OPC density (Pdgfrα^+^/mm^2^) in the hippocampus [(c): two‐way ANOVA, genotype: *F* (1, 23) = 1.938, *p* = .1772; age: *F* (2, 23) = 0.3121, *p* = .7349; interaction: *F* (2, 23) = 2.015, *p* = .1562], the entorhinal cortex [(d): two‐way ANOVA, genotype: *F* (1, 25) = 1.188, *p* = .2861; age: *F* (2, 25) = 1.162, *p* = .3292; interaction: *F* (2, 25) = 3.189, *p* = .0584] and the fimbria [(e): two‐way ANOVA, genotype: *F* (1, 23) = 0.5396, *p* = .4700; age: *F* (2, 23) = 12.37, *p* = .0002; interaction: *F* (2, 23) = 0.2118, *p* = .8107] of control (open circles, black bars) and *MAPT^P301S^* (black squares, open bars) mice. (f‐k) Representative confocal image stacks showing PDGFRα (red), EdU (green) and Hoescht 33342 (blue) labelling in the hippocampus (f‐g), entorhinal cortex (h‐i) and fimbria (j‐k) of P180 wild‐type (WT; open circles, black bars) and *MAPT^P301S^* (black squares, open bars) mice following 5 days of EdU administration. (l) Quantification of the proportion of EdU^+^ OPCs (EdU^+^ PDGFRα^+^/PDGFRα^+^) in the hippocampus, entorhinal cortex and fimbria of WT and *MAPT^P301S^* mice [(l): two‐way ANOVA, genotype: *F* (1, 20) = 11.98, *p* = .0025; brain region: *F* (2, 20) = 121.7, *p* < .0001; interaction: *F* (2, 20) = 5.997, *p* = .0091]. (m‐n) Representative confocal images showing cells expressing PDGFRα (red) and Ki67 (green) in the fimbria of P180 WT (m) and *MAPT^P301S^* (n) mice. (o) Quantification of the proportion of Ki67^+^ OPC (Ki67^+^ PDGFRα^+^/PDGFRα^+^) at P180 within the fimbria (two‐tailed, unpaired *t* test, *p* = .798). Data are presented as mean ± *SD* for *n* = 3–6 mice per group. Arrows indicate PDGFRα^+^ OPCs. Arrow heads indicate recently divided OPCs (EdU^+^ PDGFRα^+^). Scale bars represent 80 µm (a‐b); 40 µm (f‐k); 20 µm (m‐n)

To determine whether human hyperphosphorylated tau influenced new oligodendrocyte number by modulating OPC proliferation, we next evaluated PDGFRα^+^ OPC density in the hippocampus (Figure 3a‐c), entorhinal cortex (Figure 3d) and fimbria (Figure 3e) of control and *MAPT^P301S^* mice at P60 + 7, P60 + 90 and P60 + 120. OPC density was equivalent in control and *MAPT^P301S^* mice (hippocampus: *p* = .17, entorhinal cortex: *p* = .28, fimbria: *p* = .47) and did not change with age in any region (Figure 3c‐e). To determine whether OPC proliferation was affected by hyperphosphorylated tau, P175 WT and *MAPT^P301S^* mice were given the thymidine‐analogue EdU via their drinking water for 5 consecutive days, and coronal cryosections processed to detect PDGFRα (red) and EdU (green) (Figure 3f‐k). The proportion of OPCs that proliferated to incorporate EdU (EdU^+^ PDGFRα^+^/PDGFRα^+^ × 100) was equivalent in the hippocampus (Figure 3f, g, l; *p* = .81) and entorhinal cortex (Figure 3h, i, l; *p* = .97) of WT and *MAPT^P301S^* mice. By contrast, we detected a small but significant increase in OPC proliferation in the fimbria of *MAPT^P301S^* mice, compared with WT mice (Figure 3j‐l; *p* = .0004), but this was not a large enough change in proliferation to overtly alter the proportion of OPCs that expressed the proliferative marker Ki67 at P180 (Figure 3m‐n; *p* = .79). Consequently, this small increase in fimbria OPC proliferation appears unable to explain the large increase in new oligodendrocyte number noted across the hippocampus, entorhinal cortex and fimbria of *MAPT^P301S^* mice at P60 + 120 (Figure 2).

### 3.4 Oligodendrocyte density is normal in MAPT^P301S^ mice

**Figure 4:**
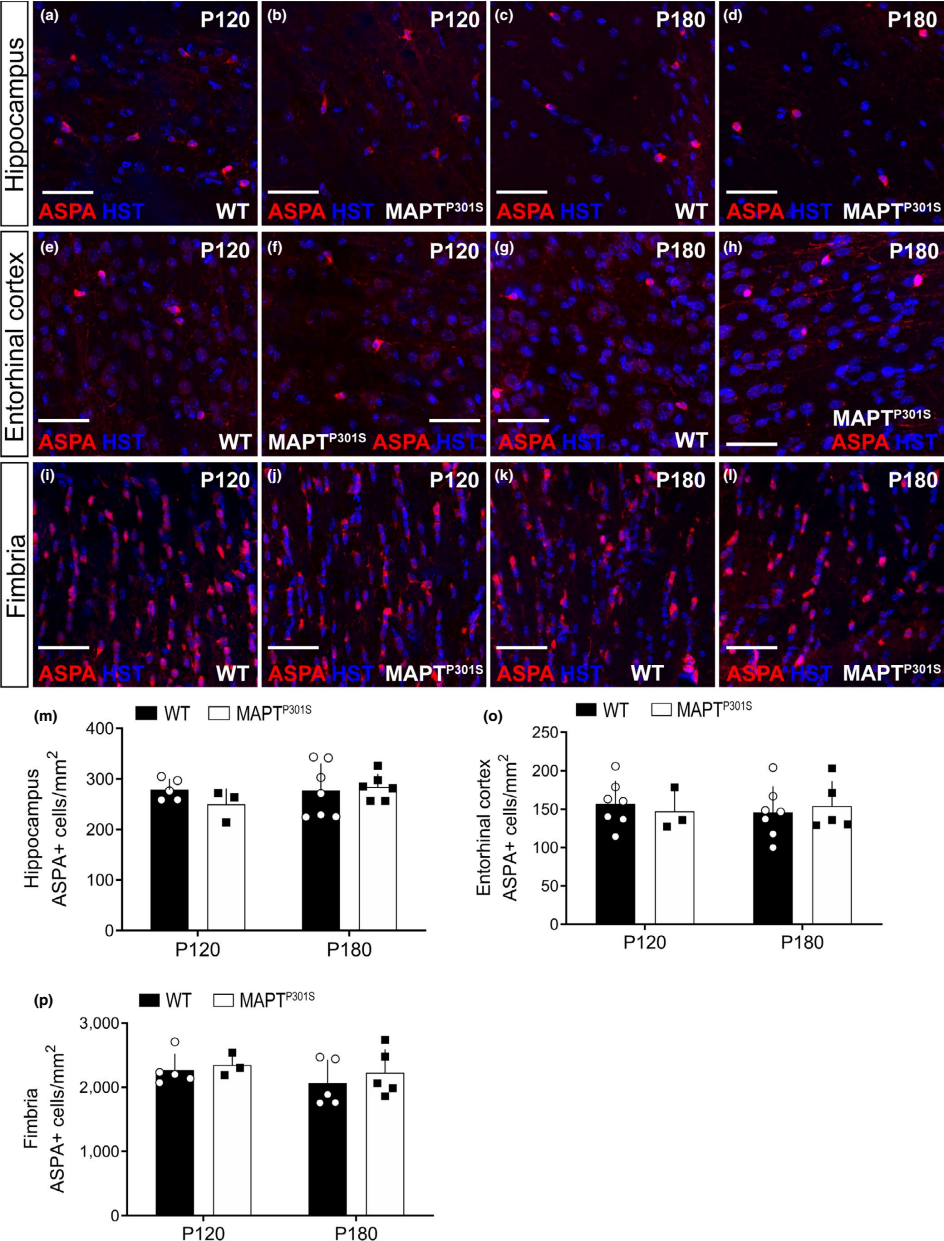
Oligodendrocyte population is not altered in *MAPT^P301S^* mice at P120 and P180. (a‐l) Representative confocal images of ASPA (red) and Hoechst 33342 (blue) in the hippocampus (a‐d), entorhinal cortex (EC; e‐h) and fimbria (i‐l) of wild‐type (WT) and *MAPT^P301S^* mice at P120 and P180. (m‐o) Quantification of total oligodendrocyte density (ASPA^+^/mm^2^) in the hippocampus [(m): two‐way ANOVA, genotype: *F* (1, 17) = 0.417, *p* = .526; age: *F* (1, 17) = 0.849, *p* = .369; interaction: (1, 17) = 1.100, *p* = .308], the entorhinal cortex [(n): two‐way ANOVA, genotype: *F* (1, 18) = 0.004, *p* = .948; age: *F* (1, 18) = 0.0254, *p* = .875; interaction: *F* (1, 18) = 0.397, *p* = .536] and the fimbria [(o): two‐way ANOVA, genotype: *F* (1, 14) = 0.594, *p* = .453; age: *F* (1, 14) = 1.145, *p* = .302; interaction: *F* (1, 14) = 0.080, *p* = .781] of WT (open circles, black bars) and *MAPT^P301S^* (black squares, open bars) mice at P120 and P180. Data are presented as mean ± *SD*, *n* = 3–5 mice per group. Scale bars represent 40 µm

To determine whether the increase in new oligodendrocyte addition was accompanied by an overall increase in oligodendrocyte number in the hippocampus (Figure 4a‐d), entorhinal cortex (Figure 4e‐h) or fimbria (Figure 4i‐l), we performed immunohistochemistry on coronal brain cryosections from P120 and P180 WT and *MAPT^P301S^* mice, to detect the oligodendrocyte marker aspartoacylase (ASPA). ASPA labels post‐mitotic oligodendrocytes that also express adenomatous polyposis coli clone 1 (CC1) (Howng et al., 2010; Madhavaro et al., 2004). We found that the density of ASPA^+^ oligodendrocytes was unchanged between P120 and P180 in the hippocampus (Figure 4m; *p* = .30), entorhinal cortex (Figure 4n; *p* = .87) or fimbria (Figure 4o; *p* = .30) of WT or *MAPT^P301S^* mice, and that the overexpression of hyperphosphorylated tau did not influence total oligodendrocyte density in any region (hippocampus *p* = .52; entorhinal cortex *p* = .94; fimbria *p* = .45). As the number of newborn oligodendrocytes added to these brain regions in *MAPT^P301S^* mice exceeds the number added to controls (Figure 2), these data suggest that new oligodendrocytes are either (i) premyelinating oligodendrocytes that do not label with ASPA and contribute myelin to the brain, or (ii) mature oligodendrocytes that are needed to maintain the density of ASPA^+^ oligodendrocytes, rather than expand the population.

**Figure 5:**
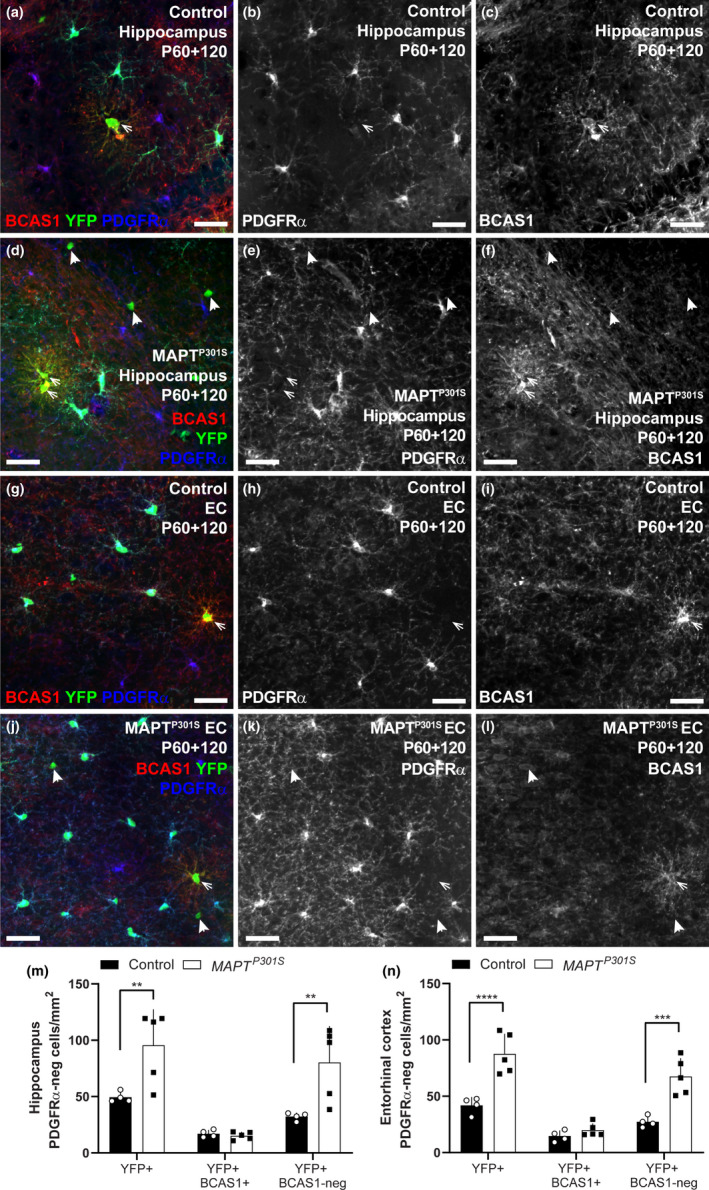
P60 + 120 *MAPT^P301S^* and control mice have a similar number of newborn BCAS1^+^ premyelinating oligodendrocytes in the hippocampus and entorhinal cortex. (a‐f) Representative confocal images showing BCAS1 (red), PDGFRα (blue) and YFP (green) in the hippocampus of P60 + 120 *Pdgfrα‐CreER^T2^::Rosa26‐YFP* (control; a‐c) and *Pdgfrα‐CreER^T2^::Rosa26‐YFP::Prnp‐MAPT^P301S^* (*MAPT^P301S^*; d‐f) mice at ~Bregma −2.7. (g‐l) Representative confocal images showing BCAS1 (red), PDGFRα (blue) and YFP (green) in the entorhinal cortex (EC) of P60 + 120 *Pdgfrα‐CreER^T2^::Rosa26‐YFP* (control; g‐i) and *Pdgfrα‐CreER^T2^::Rosa26‐YFP::Prnp‐MAPT^P301S^* (*MAPT^P301S^*; j‐l) mice at ~Bregma −2.7. (m) Quantification of the density of all YFP^+^ cells that are PDGFRα‐negative in the hippocampus of P60 + 120 control and *MAPT^P301S^* mice, including those that are YFP^+^ PDGFRα‐negative BCAS1^+^ premyelinating oligodendrocytes versus YFP^+^ PDGFRα‐negative BCAS1‐negative mature oligodendrocytes. [two‐way ANOVA, genotype: *F* (1, 21) = 15.73, *p* = .0007; cell type: *F* (2, 21) = 18.59, *p* < .0001; interaction: *F* (2, 21) = 4.428., *p* = .024]. (n) Quantification of the density of all YFP^+^ cells that are PDGFRα‐negative in the entorhinal cortex of P60 + 120 control and *MAPT^P301S^* mice, including those that are YFP^+^ PDGFRα‐negative BCAS1^+^ premyelinating oligodendrocytes versus YFP^+^ PDGFRα‐negative BCAS1‐negative mature oligodendrocytes. [two‐way ANOVA, genotype: *F* (1, 21) = 46.37, *p* < .0001; cell type: *F* (2, 21) = 38.57, *p* < .0001; interaction: *F* (2, 21) = 8.00, *p* = .002]. Asterisks indicate significant differences identified by Bonferroni post hoc analysis, **p < .01, ***p < .001, ****p < .0001. Scale bars represent 35 µm

To determine whether the newborn oligodendrocytes added to the brains of P60 + 120 control (*Pdgfrα‐CreER^T2^:: Rosa26‐YFP*) and *MAPT^P301S^* (*Pdgfrα‐CreER^T2^::Rosa26‐YFP::Prnp*‐*MAPT^P301S^*) mice are immature or mature oligodendrocytes, coronal cryosections from ~Bregma −2.7, containing the hippocampus (Figure 5a‐f) and entorhinal cortex (Figure 5g‐l), were stained to detect PDGFRα^+^ (blue), YFP (green) and Breast Carcinoma Amplified Sequence 1 (BCAS1, red), a marker of immature, premyelinating oligodendrocytes (Fard et al., 2017). Consistent with our previous data, the density of new oligodendrocytes (YFP^+^, PDGFRα‐negative cells) in the hippocampus (Figure 5m; *p* = .0075) and entorhinal cortex (Figure 5n; *p* < .0001) was significantly higher in P60 + 120 *MAPT^P301S^* mice compared with controls; however, the density of new YFP^+^ oligodendrocytes that labelled with BCAS1, identifying them as premyelinating cells, was unaltered (Figure 5m‐n; hippocampus, *p* > .99; entorhinal cortex, *p* > .99), suggesting that the overexpression of hyperphosphorylated tau was not associated with the addition of more premyelinating cells, but was instead associated with an increase in the addition of mature oligodendrocytes.

**Figure 6:**
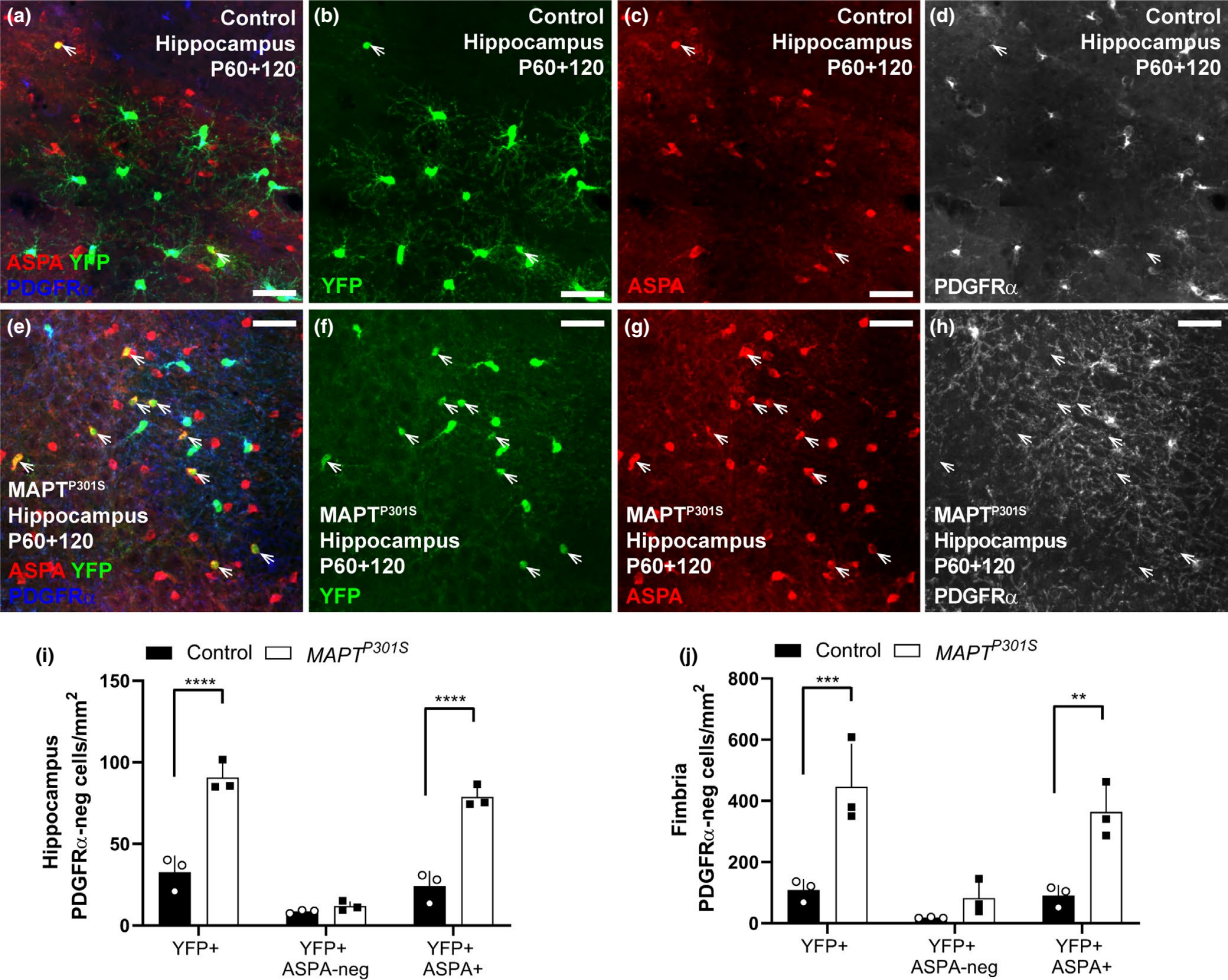
*MAPT^P301S^* mice add more ASPA^+^ mature oligodendrocytes to the hippocampus and fimbria than control mice between 5 and 6 months of age. (a‐h) Confocal images showing ASPA (red), PDGFRα (blue) and YFP (green) in the hippocampus of P60 + 120 *Pdgfrα‐CreER^T2^:: Rosa26‐YFP* (control; a‐d) and *Pdgfrα‐CreER^T2^:: Rosa26‐YFP:: Prnp‐MAPT^P301S^* (*MAPT^P301S^*; e‐h) mice at ~Bregma −1.7. (m) Quantification of the density of all YFP^+^ cells that are PDGFRα‐negative in the hippocampus of P60 + 120 control and *MAPT^P301S^* mice, including those that are YFP^+^ PDGFRα‐negative ASPA‐negative premyelinating oligodendrocytes versus YFP^+^ PDGFRα‐negative ASPA^+^ mature oligodendrocytes. [two‐way ANOVA, genotype: *F* (1, 12) = 120.5, *p* < .0001; cell type: *F* (2, 12) = 79.72, *p* < .0001; interaction: *F* (2, 12) = 25.19, *p* < .0001]. (n) Quantification of the density of all YFP^+^ cells that are PDGFRα‐negative in the fimbria of P60 + 120 control and *MAPT^P301S^* mice, including those that are YFP^+^ PDGFRα‐negative ASPA‐negative premyelinating oligodendrocytes versus YFP^+^ PDGFRα‐negative ASPA^+^ mature oligodendrocytes. [two‐way ANOVA, genotype: *F* (1, 12) = 40.81, *p* < .0001; age: *F* (2, 12) = 15.27, *p* = .0005; interaction: *F* (2, 12) = 5.46, *p* = .02]. Asterisks indicate significant differences identified by Bonferroni post hoc analysis, **p < .01, ***p < .001, ****p < .0001. Scale bars represent 35µm

To further confirm this, we immunolabelled coronal cryosections at ~Bregma −1.7, containing the hippocampus and fimbria, to detect PDGFRα^+^ (blue), YFP (green) and ASPA (red) (Figure 6a‐h). In the hippocampus (Figure 6i) and fimbria (Figure 6j), we detected a small population of YFP^+^ presumptive premyelinating cells (PDGFRα‐neg, ASPA‐neg) and the density of these cells was equivalent between control and *MAPT^P301S^* mice. However, the density of YFP^+^ ASPA^+^ mature oligodendrocytes was significantly increased in *MAPT^P301S^* mice compared with controls, in both regions (Figure 6i‐j; hippocampus, *p* < .0001; fimbria, *p* = .0023). As 284 ± 36 mature oligodendrocytes (mean ± *SD*) are present per mm^2^ of hippocampus, and 79 ± 6 cells/mm^2^ are newborn (mean ± *SD*), this equates to ~27% of oligodendrocytes present in the P180 *MAPT^P301S^* hippocampus being newborn. In the entorhinal cortex and fimbria, it equates to ~44% and 16%, respectively. As the new oligodendrocytes comprise such a large proportion of all oligodendrocytes in the hippocampus, entorhinal cortex and fimbria of the P180 *MAPT^P301S^* mice, but do not increase total oligodendrocyte density beyond that seen in WT mice, it is likely that oligodendrocyte addition is accompanied by oligodendrocyte loss in the *MAPT^P301S^* mouse brain.

### 3.5 Newly myelinated axons are more abundant in MAPT^P301S^ mice

**Figure 7:**
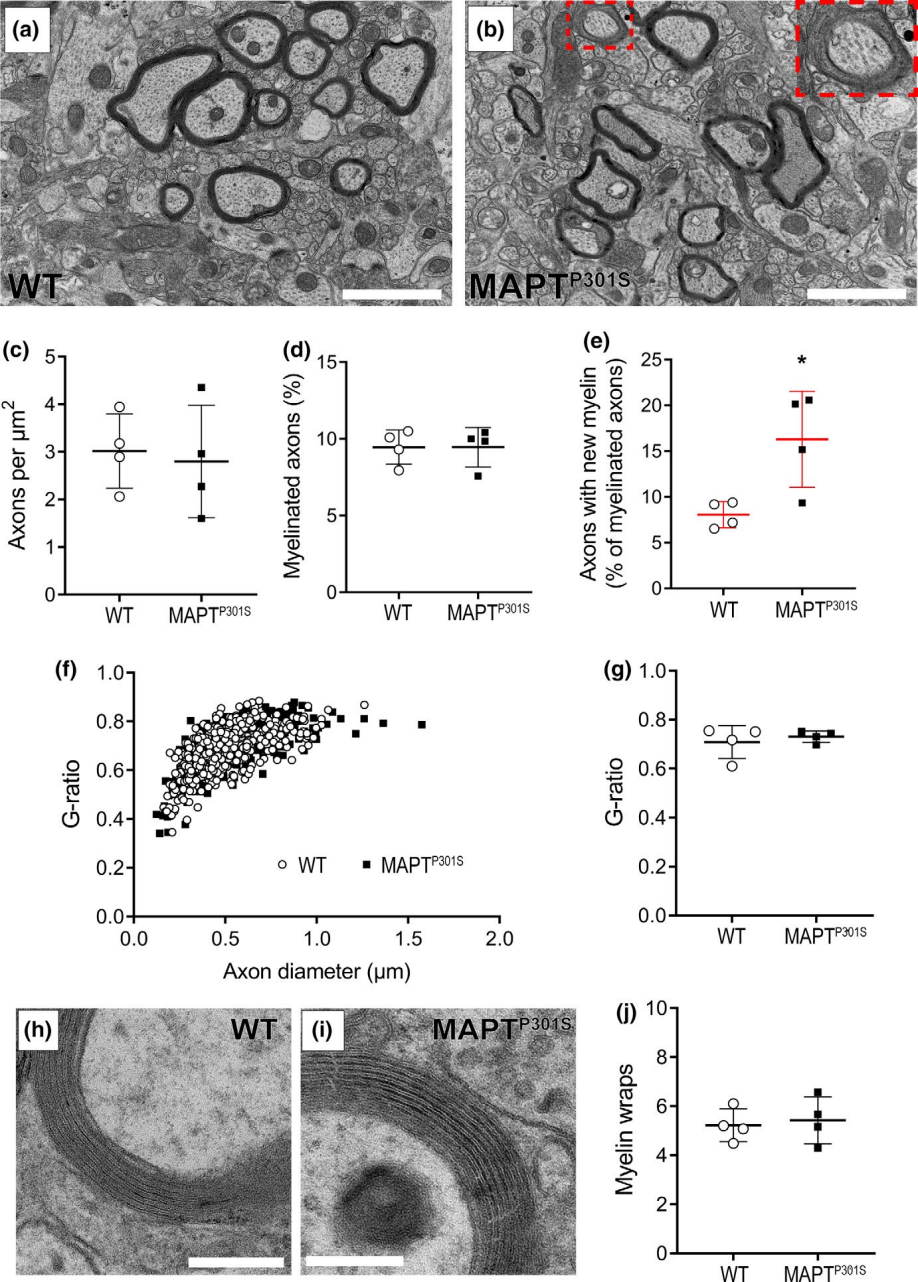
The proportion of newly myelinated axons is increased in *MAPT^P301S^* mice at P180. (a‐b) Representative electron micrographs from the CA1 region of the hippocampus of WT (a) and *MAPT^P301S^* (b) mice at P180. Red box and inset (b) show a newly myelinated axon (c) Quantification of axon density (axons/µm^2^) in WT (open circles) and *MAPT^P301S^* (black squares) mice (two‐tailed *t* test, *t* = 0.3133, *df* = 6, *p* = .7647). (d) Quantification of the proportion of myelinated axons in the CA1 of WT and *MAPT^P301S^* mice at P180 (two‐tailed *t* test, *t* = 0.003, *df* = 6 *p* = .9970). (e) Quantification of the proportion of myelinated axons ensheathed by immature (new) myelin (two‐tailed *t* test, *t* = 3.027, *df* = 6, *p* = .0232). (f) Graphical representation of the g‐ratio distribution based on axon diameter (Simple linear regression analysis, slope *F* (1, 1,062) = 1.344, *p* = .2466, Y intercept *F* (1, 1,063) = 0.1533, *p* = .6955 *n* = 55–190 myelinated axons per mouse). (g) Quantification of average g‐ratio per animal in WT and *MAPT^P301S^* mice at P180 (two‐tailed *t* test, *t* = 0.6236, *df* = 6, *p* = .5558). (h‐i) Representative high magnification electron micrographs through a single myelinated axon within the CA1 region of the number of myelin wraps in WT (h) and MAPT mice (i) at P180. (j) Quantification of average myelin wraps per animal in WT and *MAPT^P301S^* mice at P180 (two‐tailed *t* test, *t* = 0.3533, *df* = 6, *p* = .7359). Scale bars represent: 1 µm (a‐b), 300 nm and 100 nm (h, i). Results are presented as mean ± *SD*, *n* = 4 mice per genotype

To further explore the possibility of oligodendrocyte turnover, we next quantified myelination in the stratum lacunosum moleculare of the CA1 region of the hippocampus (Figure 7) and in the fimbria (Fig. S5) of P180 WT and *MAPT^P301S^* mice by transmission electron microscopy. We found that in both regions, total axon density (Figure 7a‐c, *p* = .76; Fig. S5a, *p* = .79) and the proportion of myelinated axons (Figure 7d, *p* = .99; Fig. S5b, *p* = .95) were equivalent between WT and *MAPT^P301S^* mice. However, looking more closely at the myelinated axons, we found that the proportion of myelinated axons that were ensheathed by immature myelin internodes was increased in both the hippocampus (Figure 7d, *p* = .02) and fimbria (Fig. S5c, *p* = .02) of *MAPT^P301S^* mice compared with WT mice. In cross section, these myelin internodes were characterised by an enlarged cytoplasmic inner tongue process located between the axon and the initial myelin wrap (red box and inset Figure 7b), indicating that myelin compaction is not yet complete (Kang et al., 2013; Liu et al., 2012). These axons represent ~16% of the myelinated axon population in the hippocampus and ~5% of myelinated axons in the fimbria of *MAPT^P301S^* mice, but do not alter the g‐ratio [axon diameter/(axon + myelin diameter)] of myelinated axons in either region (hippocampus: Figure 7e‐I; fimbria: Fig. S5d), or the average g‐ratio per mouse (Figure 7j, *p* = .55; Fig. S5e, *p* = .25). The average number of myelin wraps per mature myelin sheath (Figure 7k, *p* = .73) was also equivalent between WT and *MAPT^P301S^* mice. As more axons were newly ensheathed in the hippocampus and fimbria of P180 *MAPT^P301S^* mice than WT mice, yet the overall level of myelination is the same, new oligodendrocytes must engage in myelin replacement in the early stages of taupathology in this preclinical model.

## 4 DISCUSSION

Myelin and axon losses are associated with cognitive decline and are exacerbated in people diagnosed with tauopathy. However, the behaviour of OPCs early in the disease process and the potential for myelin repair remain unclear. Herein, by tracing the fate of OPCs in *MAPT^P301S^* transgenic mice, we show that the number of adult‐born oligodendrocytes increases in the hippocampus, entorhinal cortex and fimbria between 5 and 6 months of age, prior to the onset of overt cognitive symptoms. This increase in new oligodendrocyte addition cannot be explained by an equivalent increase in OPC proliferation and was not associated with a change in total oligodendrocyte density, or the number of axons that were myelinated within the hippocampus or fimbria; however, a larger proportion of the axons were ensheathed by immature myelin internodes, which may be indicative of myelin repair.

### 4.1 MAPT^P301S^ transgenic mice do not develop overt motor or cognitive deficits by 6 months of age

*MAPT^P301S^* transgenic mice overexpress the T34 isoform of human MAPT (1N4R) with the P301S mutation under the regulation of the mouse prion promotor, resulting in human tau protein being expressed in the brain at levels that are 5 times higher than the endogenous mouse protein (Yoshiyama et al., 2007). To determine whether the relative expression of human tau protein changed with age in the hippocampus, we performed western blot analysis of hippocampal lysates from P30, P60, P90 and P180 mice. We found that human tau and hyperphosphorylated human tau were not detected in WT mice, but were abundantly expressed in *MAPT^P301S^* mice, and that their relative expression levels remained stable over time (Figure 1). When *MAPT^P301S^* mice are maintained on a B6C3H genetic background, they exhibit prominent microglial activation from 3 months of age, prior to the formation of neurofibrillary tangles at 6 months of age (Yoshiyama et al., 2007). However, when we maintain the *MAPT^P301S^* mice on a C57BL/6 background, we detect microglial activation at 6 months of age (Fig. S2), and they develop neurofibrillary tangles at 10 months of age (Dumont et al., 2011), suggesting that the genetic background of these mice can significantly impact the time course of their pathology. The more severe phenotype of *MAPT^P301S^* mice on a B6C3H background is further supported by poorer survival outcomes with ~25% dying by 6 months of age (Yoshiyama et al., 2007) compared with only ~15% of mice dying within the first 6 months when they are crossed onto a C57BL/6 background (Merchán‐Rubira et al., 2019; our Fig. S1).

Tauopathies are associated with progressive motor degeneration and cognitive impairment (Lewis et al., 2000; Ramsden et al., 2005; Santacruz et al., 2005; Yoshiyama et al., 2007; Takeuchi et al., 2011; reviewed by Ferrer, 2018). To determine whether *MAPT^P301S^* mice had impaired locomotor or cognitive performance, they were subjected to a battery of behavioural tests at 2, 3 and 6 months of age. We found that *MAPT^P301S^* mice displayed normal locomotion in an open field task, at least until 6 months of age (Figure 1), suggesting there was no overt impairment in motor function. *MAPT^P301S^* overexpression also had no effect on working memory performance in the T‐maze, short‐term recognition memory in the novel object recognition task, or spatial learning and memory in the Barnes maze at any age examined (Figure 1), suggesting that these mice do not exhibit a decline in cognitive performance until after 6 months of age. Overexpression of the P301L mutation of human *MAPT*, under the mouse prion promotor, results in a rapid decline in motor function from as early as 4.5 months of age in homozygous and 6.5 months in heterozygous mice (Lewis et al., 2000). However, consistent with our data, motor decline is not observed in mice overexpressing the P301S mutation under the same promotor (Dumont et al., 2011; Takeuchi et al., 2011; Chalermpalanupap et al., 2017; our Figure 1). Instead, transient increases in locomotor behaviour in an open field arena have been reported that are either only seen between 30 and 90 min of a 2‐hr observation time (Takeuchi et al., 2011), or present at 7 months of age but not at 10 months (Dumont et al., 2011). For *MAPT^P301S^* mice raised on the B6CH3 background, deficits in spatial memory and contextual fear conditioning have been reported at 6 (Takeuchi et al., 2011) and 7.5 (Lasagna‐Reeves et al., 2016) months of age. However, consistent with our data, other studies examining *MAPT^P301S^* mice on a C57BL/6 background show that the onset of memory impairment is delayed until after 10 months of age (Chalermpalanupap et al., 2017; Dumont et al., 2011), such that their performance in a Barnes maze is equivalent to WT mice at 6 months of age (Takeuchi et al., 2011). Our data provide further support that *MAPT^P301S^* mice maintained on a C57BL/6 background do not exhibit overt motor impairment or cognitive decline by 6 months of age. Therefore, cellular changes identified ≤6 months of age can be considered pre‐symptomatic in these mice.

### 4.2 The number of new oligodendrocytes added to the brain of MAPT^P301S^ mice increases between 5 and 6 months of age

OPCs continue to generate new oligodendrocytes at different rates in the adult mouse brain grey and white matter (Dimou, Simon, Kirchhoff, Takebayashi, & Gotz, 2008; Fukushima et al., 2015; Hill, Patel, Medved, Reiss, & Nishiyama, 2013; Rivers et al., 2008; Young et al., 2013). While the rate of OPC proliferation and oligodendrocyte addition slows with ageing in the mouse CNS (reviewed by Wang & Young, 2014), experimental interventions that produce demyelination have been shown to stimulate OPC proliferation and result in the rapid replacement of oligodendrocytes and remyelination (Assinck et al., 2017; Baxi et al., 2017; Tripathi et al., 2010; Zawadzka et al., 2010). To determine whether overexpression of the human *MAPT^P301S^* variant in mice was associated with a change in OPC behaviour and new oligodendrocyte addition, we fluorescently labelled OPCs and followed their fate overtime (Figure 2; Fig. S4). Essentially, all of the cells that became YFP‐labelled were of the oligodendrocyte lineage (Fig. S4) confirming that newly differentiated OPCs (YFP^+^ PDGFRα‐negative) were newborn oligodendrocytes. We found that a small number of newborn YFP‐labelled oligodendrocytes accumulated in the hippocampus, entorhinal cortex and fimbria of control and *MAPT^P301S^* transgenic mice between P60 and P150, and that the rate of oligodendrocyte addition during this time period was not affected by *MAPT^P301S^* expression. However, between P150 and P180, when oligodendrocyte addition was negligible in control mice, the number of YFP‐labelled oligodendrocytes increased significantly in the *MAPT^P301S^* transgenic mice, in each of the brain regions examined. Previous studies have not examined oligodendrogenesis in *MAPT^P301S^* transgenic mice; however, there is some evidence that oligodendrogenesis is increased in P60 adult *Thy1.2‐MAPT^P301S^* transgenic mice following toxin‐induced focal demyelination of the ventral funiculus in the spinal cord (Ossola et al., 2016). Fourteen days after demyelination, the density of APC^+^ OLIG2^+^ oligodendrocytes was increased in the lesion site of *Thy1.2‐MAPT^P301S^* transgenic mice compared with WT lesioned mice (Ossola et al., 2016), confirming the capacity for OPCs to efficiently remyelinate the injured CNS in the early stages of tau pathology.

A large increase in oligodendrocyte generation is often accompanied by an increase in OPC proliferation, as OPC differentiation stimulates the proliferation of adjacent OPCs to sustain the OPC population (Hughes, Kang, Fukaya, & Bergles, 2013). To determine whether expression of the human *MAPT^P301S^* variant, and the associated increase in new oligodendrocyte number, was associated with elevated OPC proliferation, dividing OPCs were EdU‐labelled in the brain of 6‐month‐old control and *MAPT^P301S^* transgenic mice (Figure 3). While OPC proliferation was elevated in the fimbria of *MAPT^P301S^* transgenic mice, when compared with the wild‐type littermates, it was not elevated in the hippocampus or entorhinal cortex. These data could be explained by an increase in oligodendrogenesis occurring close to P150, such that OPC proliferation has returned to normal by P180. Alternatively, we have previously shown that new oligodendrocyte number can be increased in response to transcranial magnetic stimulation, by enhancing the survival of the newborn cells (Cullen et al., 2019). Increased adenosine triphosphate production, through the availability of creatine, can also enhance oligodendrocyte survival during inflammation or after demyelination (Chamberlain, Chapey, Nanescu, & Huang, 2017). Therefore, it is possible that oligodendrocyte loss or other stimuli could enhance newborn oligodendrocyte survival in the *MAPT^P301S^* transgenic mice. Indeed, a combination of increased cell generation and improved survival would likely be needed to account for the substantial increase in new oligodendrocyte number observed over a one‐month period in the *MAPT^P301S^* transgenic mice.

### 4.3 Is oligodendrocyte turnover increased in MAPT^P301S^ transgenic mice?

To determine whether the large number of new oligodendrocytes added to the hippocampus, entorhinal cortex and fimbria of *MAPT^P301S^* mice increased the total number of oligodendrocytes, or acted to replace oligodendrocytes lost to pathology, we quantified ASPA^+^ oligodendrocyte density (Figure 4) as well as the proportion of newborn oligodendrocytes that were immature (BCAS1^+^/ASPA‐negative) or mature (ASPA+/ BCAS1‐negative) oligodendrocytes in each region (Figure 5 and Figure 6). Overall, oligodendrocyte density was significantly higher in the fimbria than in the hippocampus or entorhinal cortex, but for each region, it was equivalent between WT and *MAPT^P301S^* transgenic mice (Figure 4). In the *MAPT^P301S^* transgenic mice, however, a larger proportion of these cells were added and matured between 5 and 6 months of age (Figures 5, 6), suggesting that oligodendrocyte replacement had occurred. To confirm that this phenotype was not associated with neuron loss, we quantified axon density and the proportion of axons that are myelinated (Figure 7) in the CA1 subfield of the hippocampus and in the fimbria and found that was also normal in 6‐month‐old *MAPT^P301S^* transgenic mice. Overall, the proportion of axons that were myelinated, and their myelin thickness was also equivalent to that of WT mice. However, *MAPT^P301S^* had approximately twice as many axons ensheathed by immature or new myelin internodes (Figure 7 and Fig. S5).

Immature myelin is characterised by the presence of a thick cytoplasmic tongue process, forming the inner layer between the axon and the first compact myelin wrap (Kang et al., 2013; Liu et al., 2012), and suggests that the new oligodendrocytes participate in de novo myelination or myelin repair within these regions. At P180, the newborn oligodendrocytes comprise a significant proportion of all oligodendrocytes detected in the grey matter regions of *MAPT^P301S^* transgenic mice, making it unlikely that they would not alter the total oligodendrocyte addition or the proportion of axons that are myelinated, unless the increase in new oligodendrocyte number and new myelin sheath addition was accompanied by oligodendrocyte death. Therefore, we suggest that oligodendrocyte addition between P150 and P180 is driven by the need for oligodendrocyte replacement in the *MAPT^P301S^* transgenic mice and propose that these oligodendrocytes act to maintain myelin at this early stage of tauopathy.

It is well established that tauopathy can directly or indirectly result in oligodendrocyte death. For example, overexpression of the *MAPT^P301L^* variant, under the mouse α‐tubulin promotor, causes the formation of coiled filamentous tau^+^ inclusions in spinal cord oligodendrocytes, subsequently leading to oligodendrocyte degeneration and the loss of their associated myelin (Higuchi et al., 2002). Additionally, spinal cord oligodendrocytes undergo apopotosis in the *Prnp‐MAPT^P301L^* mouse model of tauopathy (Zehr et al., 2004). As oligodendrocytes express tau (Cullen et al., 2019; Young et al., 2013), it is possible that oligodendrocytes undergo apopotosis due to a direct effect of hyperphosphorylated tau; however, in the *Prnp‐MAPT^P301L^* and *MAPT^P301S^* mouse models of tauopathy, oligodendrocyte death is more likely a secondary consequence of tauopathy, as the expression of hyperphosphorylated tau is directed by the prion protein promotor in both instances. The mRNA encoding for the prion protein is expressed by nearly all cells within the mouse brain (Hrvatin et al., 2018; Zhang et al., 2014); however, when the mouse prion promotor is used to drive transcription of a reporter protein (e.g., LacZ), expression is primarily detected in neuronal populations, including hippocampal pyramidal and granule neurons that project through the fimbria (Bailly et al., 2004), and is detected in very few cells within white matter regions (Tremblay et al., 2007) where oligodendrocytes are concentrated.

Other models of neurodegenerative disease have also demonstrated increased oligodendrocyte turnover prior to the onset of disease symptoms (Behrendt et al., 2013; Desai et al., 2009, 2010; Dong et al., 2018; Kang et al., 2013). For example, oligodendrocyte loss and myelin abnormalities are observed by 6 months of age, prior to the onset of amyloid pathology, in mouse models of familial Alzheimer's disease that overexpress human mutations in the amyloid precursor and presenilin 1 proteins, either alone (APP/PS1 mice; Behrendt et al., 2013; Dong et al., 2018) or in combination with the MAPT^P301L^ mutation (3xTg mice; Desai et al., 2010; Desai et al., 2009). This was often accompanied by an increase in OPC number and oligodendrocyte generation at the same age, suggesting recruitment of these cells to repair myelin damage early in disease (Behrendt et al., 2013; Desai et al., 2010; Dong et al., 2018). Similarly, in a SOD1 mouse model of amyotrophic lateral sclerosis, oligodendrocyte loss occurs prior to the onset of disease symptoms and coincides with increased oligodendrogenesis, and acts to maintain a consistent population of oligodendrocytes within the spinal cord through to the end stage of disease (Kang et al., 2013). Consistent with our data, increased oligodendrocyte turnover was accompanied by an increase in the proportion of axons that were ensheathed by immature myelin, suggesting that these axons had been remyelinated (Kang et al., 2013). Collectively, these data indicate that oligodendrocyte turnover is a common early feature of neurodegenerative diseases associated with abnormal protein aggregation, suggesting that oligodendrocytes are highly susceptible to disease‐related stresses prior to the onset of disease symptoms. We propose that therapeutic approaches being developed to promote oligodendrocyte survival or enhancement of myelin repair for diseases such as multiple sclerosis may also be beneficial in the early stages of other neurodegenerative diseases such as tauopathies.

## CONFLICT OF INTERESTS

The authors have no competing interests to declare.

## AUTHOR CONTRIBUTIONS

KMY and CLC developed the project. SF, KAP, BSS, SW, KMY and CLC carried out the experiments. KMY, KAP and CLC obtained the funding. SF, KAP, CLC and KMY performed the statistical analyses and generated the figures. KMY, KAP and CLC provided supervision. SF, KMY, CLC and KAP wrote the paper.

## Supporting information


Figure S1.
Figure S2.
Figure S3.
Figure S4.
Figure S5.

## Data Availability

All data will be made available upon reasonable request.
