# Supplementary material for: Oligodendrogenesis increases in hippocampal grey and white matter prior to locomotor or memory impairment in an adult mouse model of tauopathy
Source: Eur J Neurosci. 2020 Apr 14;54(5):5762–84. doi: 10.1111/ejn.14726 (PMC8451881; doi:10.1111/ejn.14726)
Supplement: Supplementary file 1 — Figure S1. Figure S2. Figure S3. Figure S4. Figure S5. [file EJN-54-5762-s001.pdf]

Supplementary Material

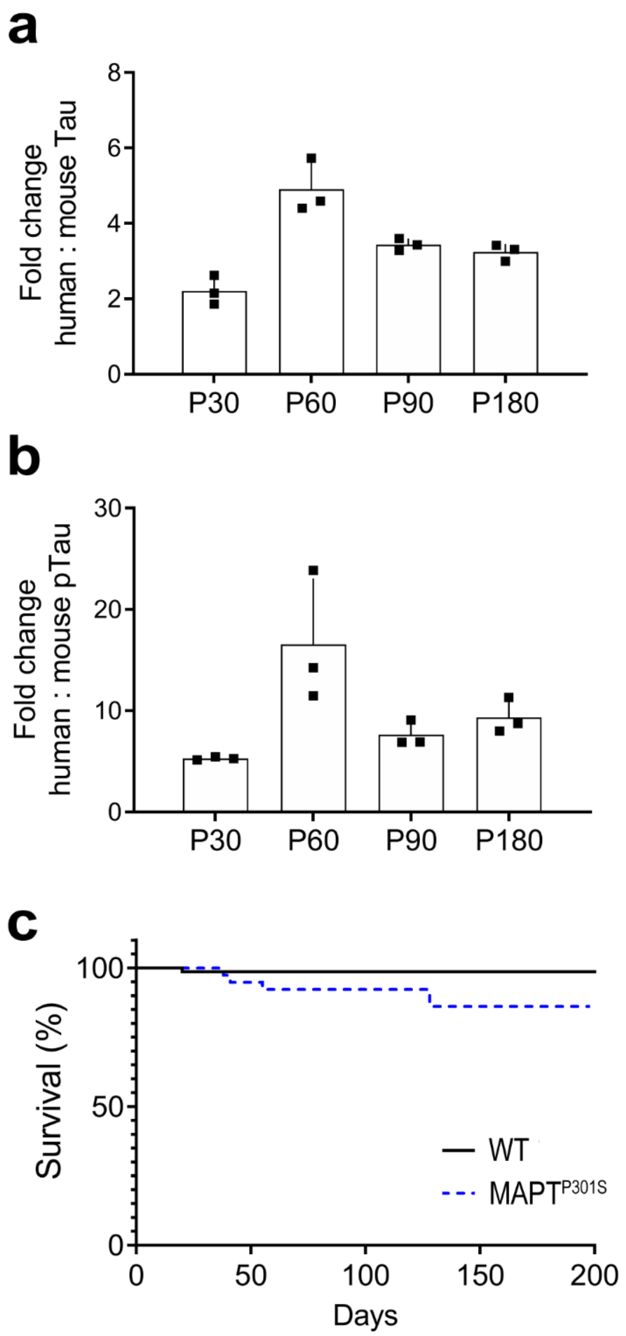

Figure S1. Human tau protein expression is 2 to 5-fold higher than endogenous mouse tau in *MAPT*^{P301S} mice

a) Fold change between human tau and mouse tau in *MAPT*^{P301S} mice at P30, P60, P90 and P180. **b**) Fold change between human phosphorylated tau and mouse phosphorylated tau in *MAPT*^{P301S} mice at P30, P60, P90 and P180. Results are presented as mean ± SD, n = 3 mice per genotype. **c**) 86% of *MAPT*^{P301S} mice (n = 39) survived to P200 against 98% of WT mice (n = 73). Survival curves comparison: Log-Rank (Mantel-Cox), p = 0.0390.

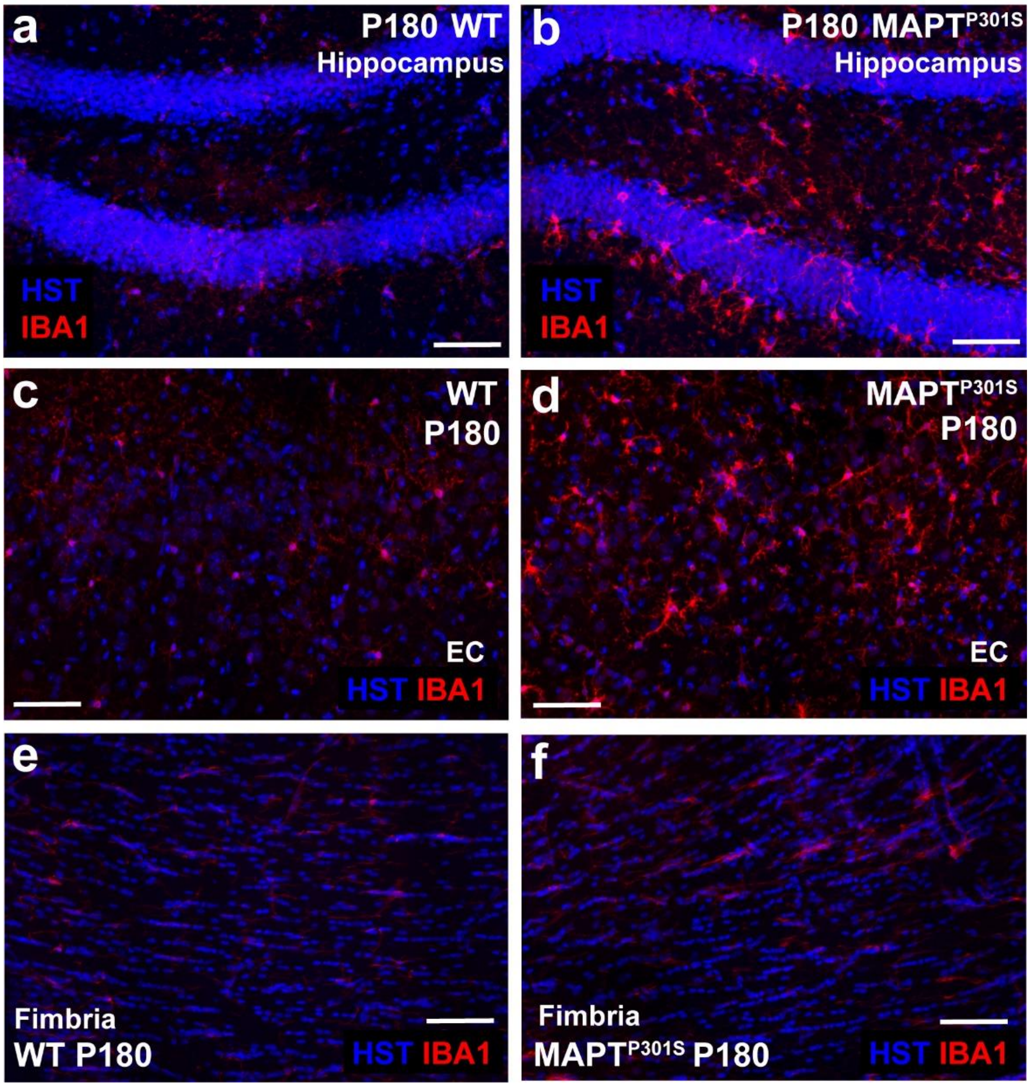

Figure S2. Reactive microglia are visible in *MAPT*^{P301S} mice at P180

a-f) Representative confocal images of Iba1 (red) and Hoechst 33342 (blue) in the hippocampus (**a-b**), entorhinal cortex (EC; **c-d**) and fimbria (**e-f**) of wildtype (**a, c, e**) and *MAPT*^{P301S} (**b, d, f**) mice at P180. Scale bars represent 60µm.

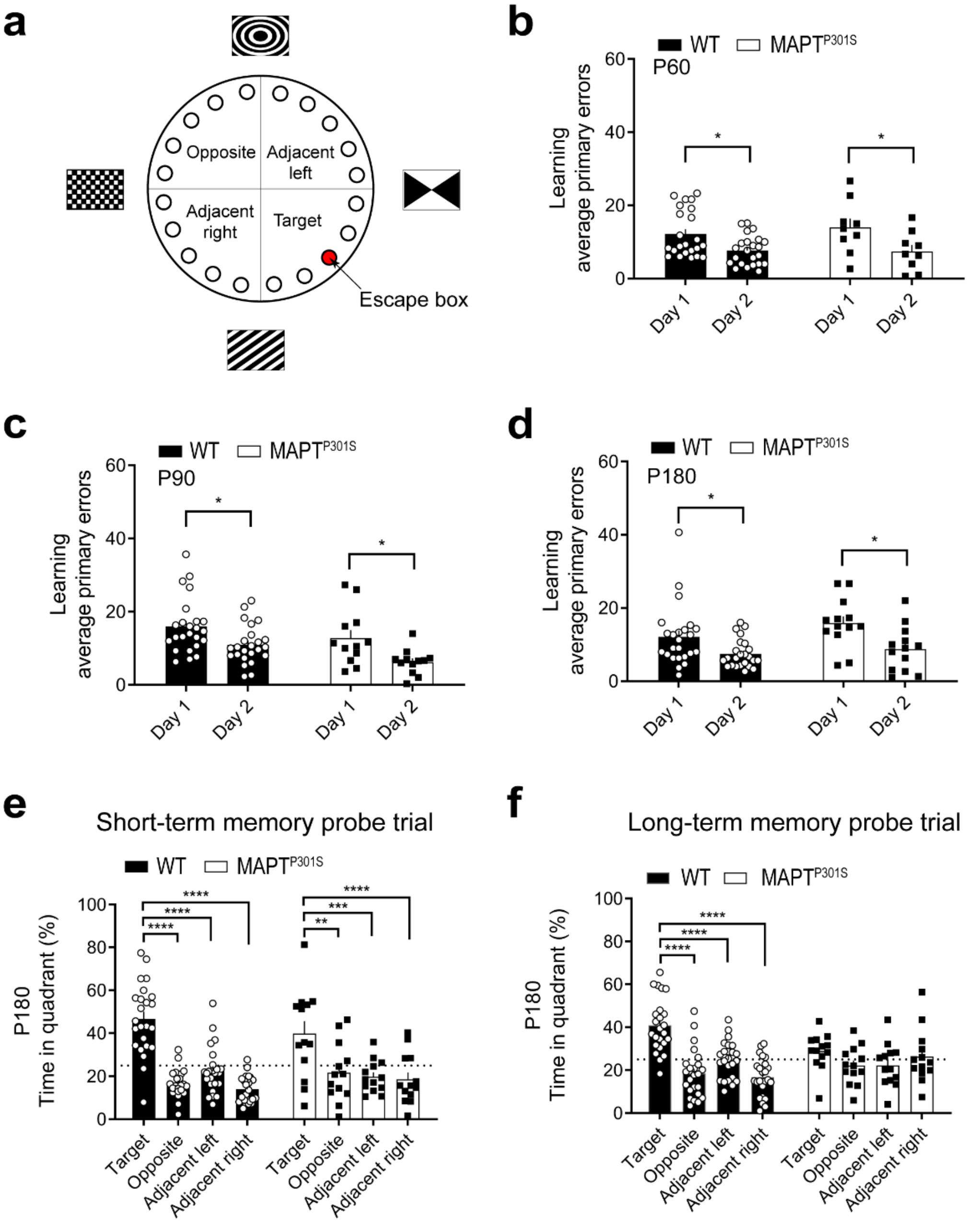

Figure S3. Learning and memory performance in the Barnes maze

a) Schematic diagram of the Barnes maze depicting the location of the escape box (red) relative to the spatial cues surrounding the maze and quadrant divisions. **b**) Quantification of the average number primary errors committed by P60 WT (open circles, black bars) and *MAPT*^{P301S} (black squares, open bars) mice during the first and second day of learning [two-way ANOVA, genotype: F (1, 60) = 0.24, p = 0.6233; learning day: F (1, 60) = 12.46, p = 0.0008; interaction: F (1, 60) = 0.39, p = 0.5307]. **c**) Quantification of the average number primary errors committed by P90 WT and *MAPT*^{P301S} mice during the first and second day of learning [two-way ANOVA, genotype: F (1, 68) = 6.30, p = 0.0144; learning day: F (1, 68) = 13.37, p = 0.0005; interaction: F (1, 68) = 0.25, p = 0.6166]. **d**) Quantification of the average number primary errors committed by P180 WT and *MAPT*^{P301S} mice during the first and second day of learning [two-way ANOVA, genotype: F (1, 70) = 2.569, p = 0.1135; learning day: F (1, 70) = 13.91, p = 0.0004; interaction: F (1, 70) = 0.5959, p = 0.4428]. **e**) Quantification of the time spent

by P180 WT and *MAPT*^{P301S} mice in each quadrant of the maze during the short-term memory probe phase [two-way ANOVA, genotype: $F(1, 140) = 6.558e\text{-}006$, $p = 0.9980$; maze quadrant: $F(3, 140) = 35.37$, $p < 0.0001$; interaction: $F(3, 140) = 1.959$, $p = 0.1230$]. **f**) Quantification of the time spent by P180 WT and *MAPT*^{P301S} mice in each quadrant of the maze during the long-term memory probe phase [two-way ANOVA, genotype: $F(1, 140) = 0.033$, $p = 0.8550$; maze quadrant: $F(3, 140) = 14.14$, $p < 0.0001$; interaction: $F(3, 140) = 6.737$, $p = 0.0003$]. Data are presented as mean ± SEM, n= 13-24 mice per group.

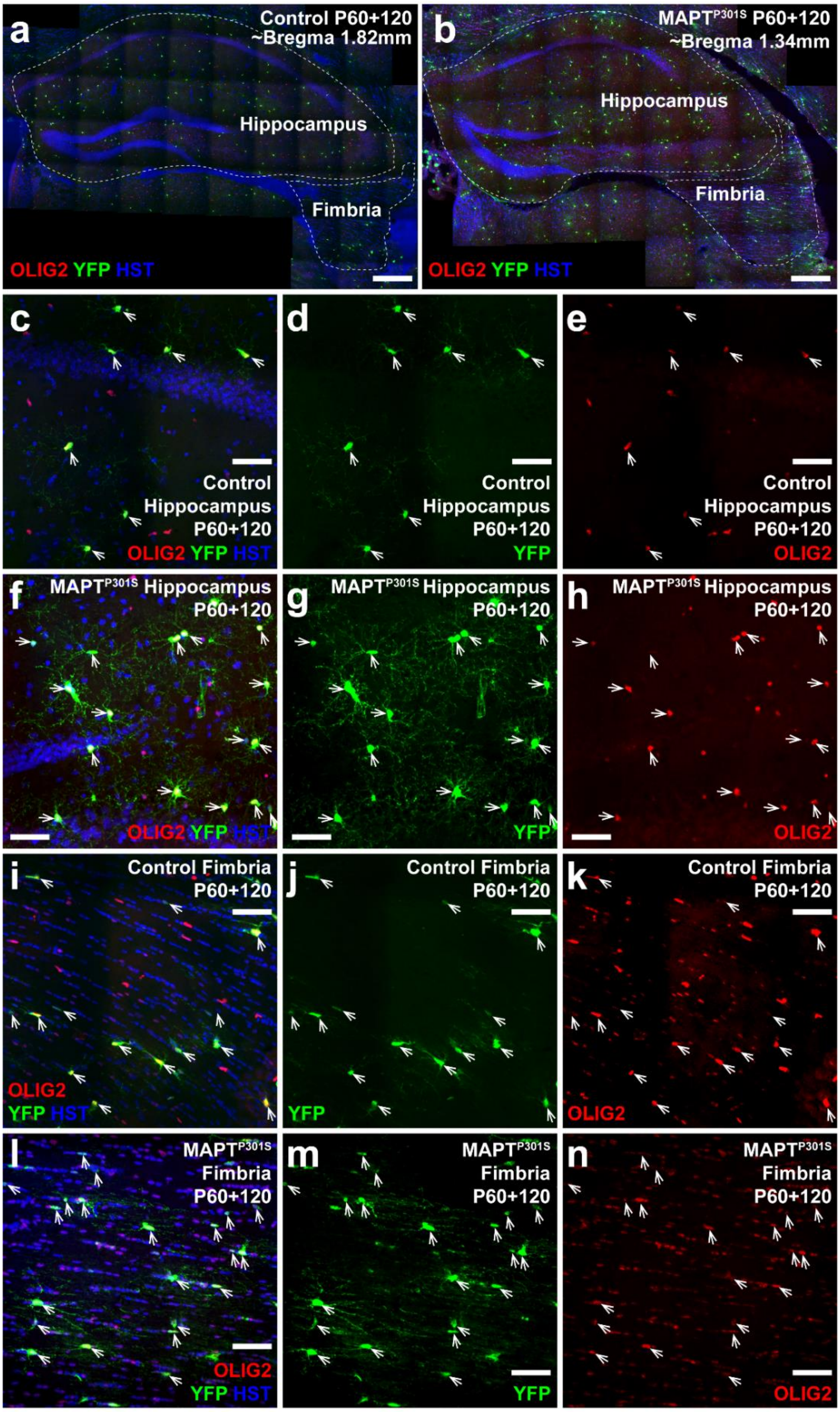

Figure S4. Essentially all YFP^{+} cells express OLIG2 in P60+120 control and *MAPT^{P301S}* mice

a-b) Low magnification confocal image showing the hippocampus and fimbria in coronal brain cryosections from P60+120 control and *MAPT^{P301S}* transgenic mice stained to detect YFP (green), OLIG2 (red) and Hoechst 33342

(blue). **c-e**) Confocal image of the hippocampus in coronal brain cryosections from P60+120 control mice stained to detect YFP (green), OLIG2 (red) and Hoechst 33342 (blue). **f-h**) Confocal image of the hippocampus in coronal brain cryosections from P60+120 $MAPT^{P301S}$ transgenic mice stained to detect YFP (green), OLIG2 (red) and Hoechst 33342 (blue). **i-k**) Confocal image of the fimbria in coronal brain cryosections from P60+120 control mice stained to detect YFP (green), OLIG2 (red) and Hoechst 33342 (blue). **l-n**) Confocal image of the fimbria in coronal brain cryosections from P60+120 *MAPT*^{P301S} mice stained to detect YFP (green), OLIG2 (red) and Hoechst 33342 (blue). White arrows indicate YFP^+ $OLIG2^+$ cells. Scale bar represents 280 µm (a-b), 50 µm (c-h), or 25 µm (i-k).

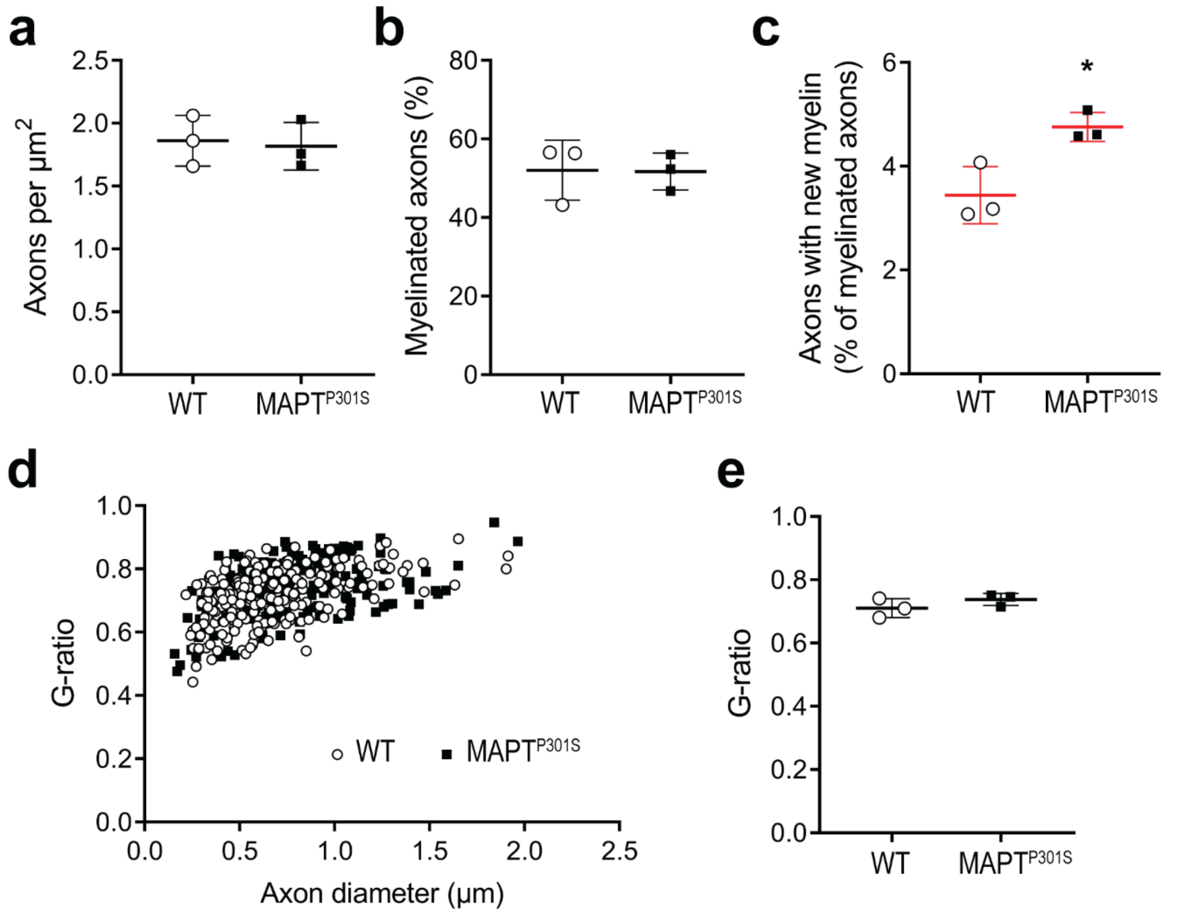

Figure S5. The proportion of newly myelinated axons is increased in the fimbria of P180 *MAPT^{P301S}* mice

a) Quantification of axon density (axons / µm^2) in the fimbria of P180 WT (open circles) and *MAPT^{P301S}* (black squares) mice (two-tailed t-test, t = 0.2750, df = 4, p = 0.7970). **d**) Quantification of the proportion of myelinated axons in the CA1 of WT and *MAPT^{P301S}* mice at P180 (two-tailed t-test, t = 0.0635, df = 4 p = 0.9524). **e**) Quantification of the proportion of myelinated axons ensheathed by immature (new) myelin (two-tailed t-test, t = 3.695, df = 4, p = 0.0209). **f**) Graphical representation of the g-ratio distribution based on axon diameter (Simple linear regression analysis, slope F (1, 524) = 1.735, p = 0.1884, Y intercept F (1, 525) = 5.422, p = 0.0203 n = 64 - 109 myelinated axons per mouse). g) Quantification of average g-ratio per animal in WT and *MAPT^{P301S}* mice at P180 (two-tailed t-test, t = 1.324, df = 4, p = 0.2562). n = 3 animals per genotype.
